# Thiodipeptides targeting the intestinal oligopeptide transporter as a general approach to improving oral drug delivery

**DOI:** 10.1016/j.ejmech.2018.06.064

**Published:** 2018-08-05

**Authors:** David W. Foley, Ravindra B. Pathak, Theresa R. Phillips, Gayle L. Wilson, Patrick D. Bailey, Myrtani Pieri, Anish Senan, David Meredith

**Affiliations:** a EPSAM Research Institute, Faculty of Natural Sciences, Keele University, Keele, Staffordshire, ST5 5BG, UK; b Department of Biological & Medical Sciences, Faculty of Health & Life Sciences, Oxford Brookes University, Gipsy Lane, Headington, Oxford OX3 0BP, UK; c Department of Life and Health Sciences, School of Sciences and Engineering, University of Nicosia, 46 Makedonitissas Avenue, CY-2417, P.O.Box 24005, CY-1700, Nicosia, Cyprus

**Keywords:** Prodrug, Membrane transporter, Intestine, PepT1, Drug delivery

## Abstract

The broad substrate capacity of the intestinal oligopeptide transporter, PepT1, has made it a key target of research into drug delivery. Whilst the substrate capacity of this transporter is broad, studies have largely been limited to small peptides and peptide-like drugs. Here, we demonstrate for the first time that a diverse range of drugs can be targeted towards transport by PepT1 using a hydrolysis resistant carrier. Eleven prodrugs were synthesized by conjugating modified dipeptides containing a thioamide bond to the approved drugs ibuprofen, gabapentin, propofol, aspirin, acyclovir, nabumetone, atenolol, zanamivir, baclofen and mycophenolate. Except for the aspirin and acyclovir prodrugs, which were unstable in the assay conditions and were not further studied, the prodrugs were tested for affinity and transport by PepT1 expressed in *Xenopus laevis* oocytes: binding affinities ranged from approximately 0.1 to 2 mM. Compounds which showed robust transport in an oocyte *trans*-stimulation assay were then tested for transcellular transport in Caco-2 cell monolayers: all five tested prodrugs showed significant PepT1-mediated transcellular uptake. Finally, the ibuprofen and propofol prodrugs were tested for absorption in rats: following oral dosing the intact prodrugs and free ibuprofen were measured in the plasma. This provides proof-of-concept for the idea of targeting poorly bioavailable drugs towards PepT1 transport as a general means of improving oral permeability.

## Highlights

• The intestinal oligopeptide transporter PepT1 is a key target for oral drug delivery.
• Non-orally bioavailable compounds conjugated to thioamides became PepT1 substrates.
• Transport of these prodrugs was shown in biological systems, including *in vivo*.
• The wide range of prodrugs synthesized demonstrates the versatility of the approach.
• Thioamide conjugation is a general means of improving poor drug oral permeability.

## Graphical abstract

**Figure:**
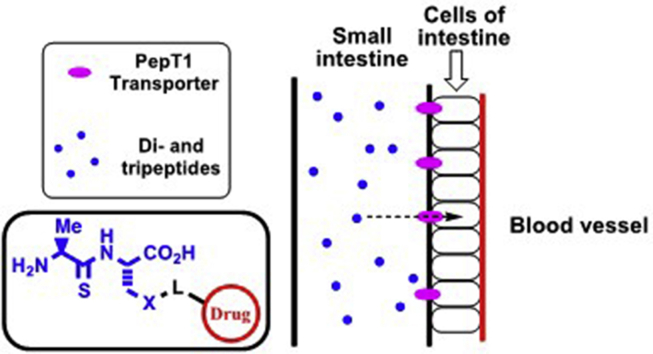

## 1 Introduction

The oral bioavailability of a compound is a crucial factor in its success or failure as a therapeutic agent, particularly given the convenience of this route of administration. There are two main mechanisms of absorption from the GI tract: passive diffusion [1] and carrier mediated transport [2]. The oral bioavailability of poorly absorbed drugs can be improved either by modifying their physicochemical properties to aid passive diffusion and/or by targeting of the compounds towards carrier mediated transport [[3], [4], [5]].

PepT1 is a proton coupled oligopeptide transporter expressed principally in the small intestine and the proximal tubule of the kidney [6]. It has a broad substrate specificity including most di- and tripeptides, β-lactam antibiotics and ACE inhibitors [7].

**Fig. 1:**
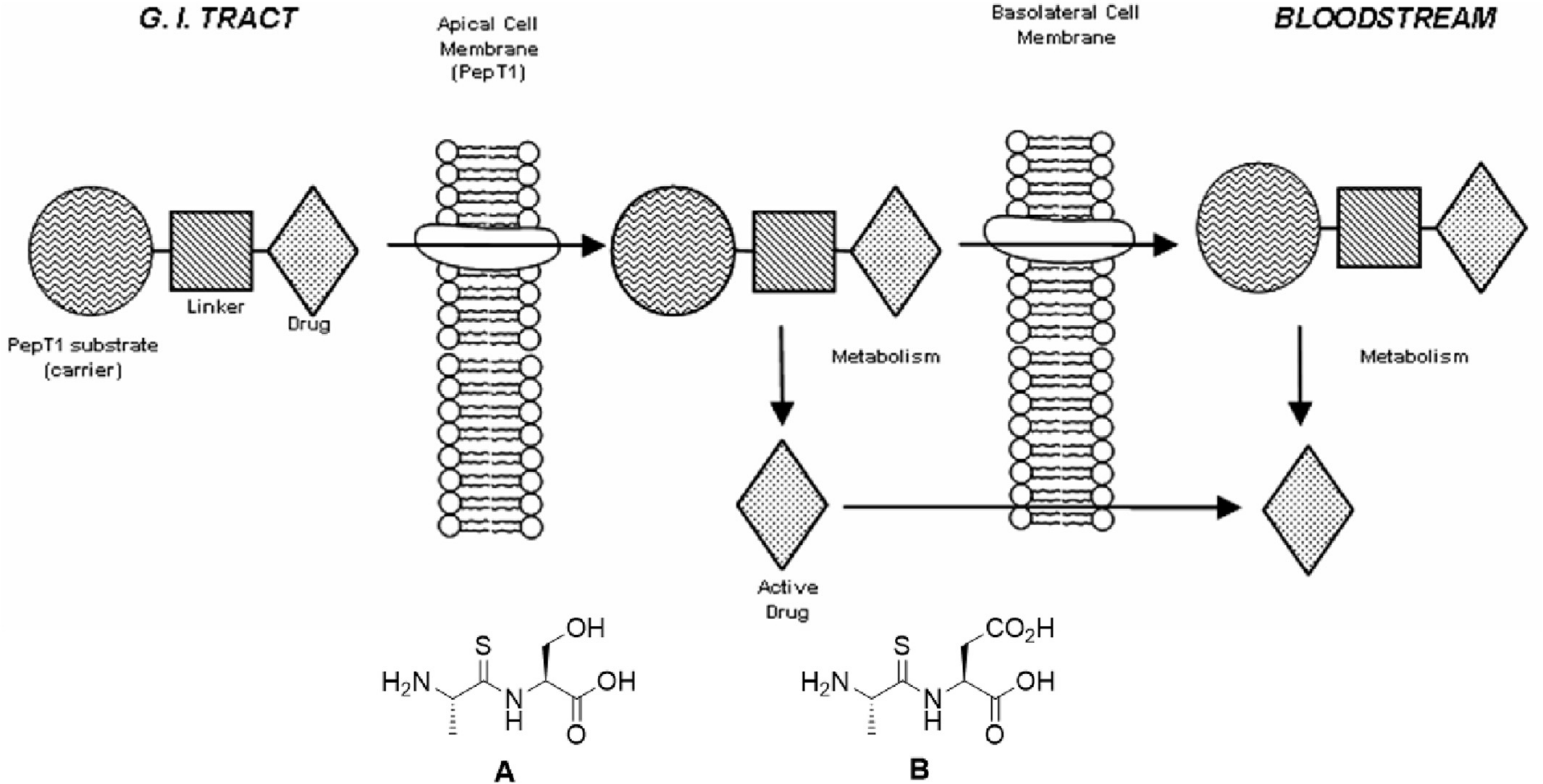
PepT1 as a drug delivery target. Drugs are attached to the side-chains of the seryl (**A**) or aspartyl (**B**) thiodipeptide carriers, directly or *via* linkers, forming prodrug substrates of PepT1.

There are many examples of targeting PepT1 to improve the oral bioavailability of pharmacologically active compounds, usually by modifying them so that they resemble the natural di- or tripeptide substrates [[8], [9], [10], [11], [12], [13]]. We have patented [14] a set of thiodipeptide substrates (such as **A** and **B**) that we hope can act as “carriers” for drug transport by PepT1 generally, and have previously published our work on model systems demonstrating that a variety of linkers can be employed [15,16]. The basic premise is illustrated in Fig. 1 in which drugs are conjugated directly or by a linker to our thiodipeptides, converting them into prodrugs that are PepT1 substrates.

**Fig. 2:**
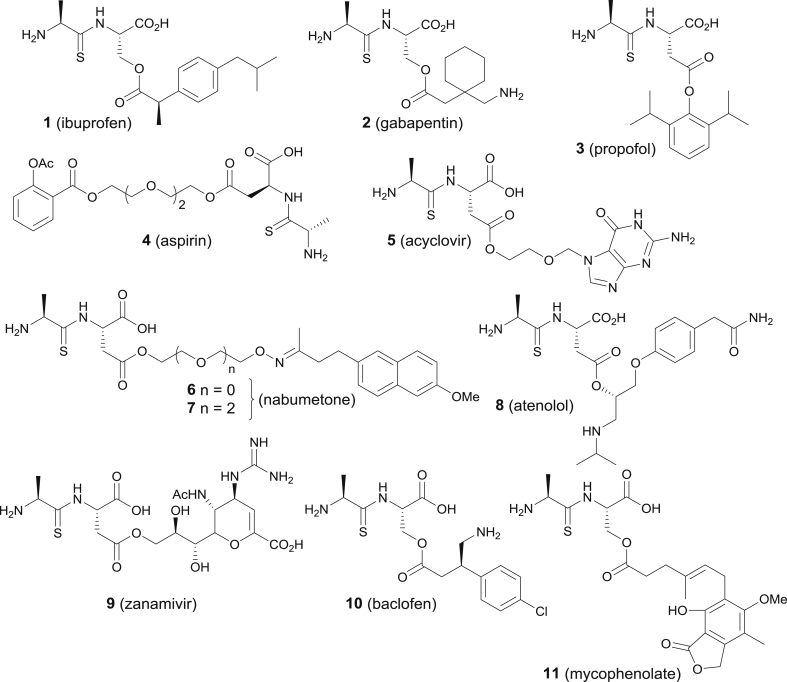
Rationally designed prodrugs to target PepT1 (parent drug in brackets).
ii) *Drugs with poor oral bioavailability*. This is a major challenge in drug development. A search of ChEMBL [18] identified several marketed drugs with low, highly variable or no oral bioavailability [17]: gabapentin (an anticonvulsant and analgesic); baclofen (a GABA receptor agonist); propofol (chemotherapeutic nausea and intractable migraine); zanamivir (treatment and prophylaxis of influenza) and mycophenolic acid (an immunosuppressant). Prodrugs **2**, **3**, **5**, and **8**–**11** (Fig. 2) were synthesized to prove our concept in this important area.

In this paper, we apply the results of our previously reported characterisation of the structure-transport relationships for PepT1 [15] to drug delivery challenges and report proof-of-concept studies that validate the use of our thiodipeptide carriers as a general approach for targeting a variety of drugs towards PepT1 mediated transport. We focused on two major areas that we felt could benefit from our thiodipeptide drug delivery technology:

i) *Drugs with GI side effects*. A common class of such drugs are the NSAIDs, as exemplified by aspirin and ibuprofen [17]. Whilst these drugs have high oral bioavailability, they also can cause severe gastric side effects. If a prodrug strategy could be developed so that bioavailability was retained, but active drug was not released close to the GI tract, such side effects might be significantly reduced. Prodrugs **1**, **4** and **6**–**7** of ibuprofen, aspirin and nabumetone respectively (Fig. 2) were synthesized to explore this area.

## 2 Chemistry

**Scheme 1:**
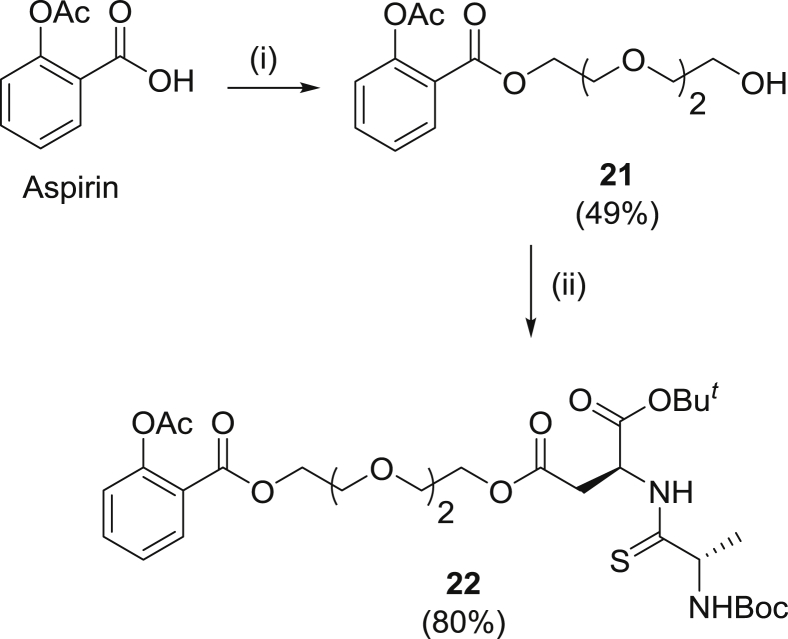
Synthesis of protected aspirin pro-drug. (i) PPh_3_, triethylene glycol, DIAD, THF, rt, sonication, 15 min (ii) **13** [15], HBTU, DIPEA, DMF, rt, 4 days.

The synthesis of the protected serine and aspartate carrier thiodipeptides (**12** and **13**), nabumetone prodrugs **6**–**7** and ibuprofen prodrug **1** have been reported previously [15,16]. Our chosen drugs could readily be attached to the appropriate carrier using standard coupling reagents, except for the aspirin prodrug **4** (Table 1). This was synthesized by first using concentrated Mitsunobu conditions [19] with sonication to esterify the salicylic acid with triethylene glycol to give **22**, then coupling this glycol ester to the aspartate carrier using standard coupling conditions (Scheme 1) to give **23**. This indirect route was chosen because we were unable to accomplish direct esterification of aspirin with the serine carrier using a variety of coupling conditions. Deprotection was usually achieved in >85% yield using either a 33% solution of TFA in DCM or neat formic acid, except for **5**, for which decomposition was avoided by using phenol as solvent [20]. Since the NMR [15] of carriers **12** and **13** show no signs of epimerisation, and rotamers observed in the NMR of some final compounds have spectral characteristics consistent with *cis*/*trans* rotamers around the thioamide bond as we have previously reported [21], we do not believe epimerisation occurred during synthesis.

**Table 1:**
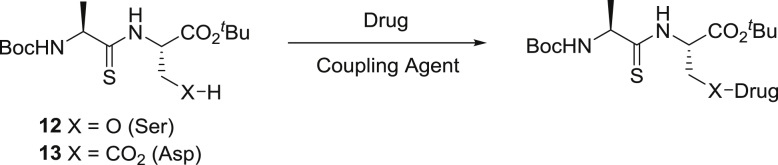
Synthesis of protected pro-drugs (non-optimized yields).

Drug	Coupling Agent	Compound (Yield)
Gabapentin (Boc protected)	HBTU	**14** (22%)
Propofol	DCC	**15** (38%)
Acyclovir	DCC	**16** (96%)
Atenolol (Boc protected)	CDI	**17** (36%)
Zanamivir	HATU	**18** (11%)
Baclofen (Boc protected)	DCC	**19** (65%)
Mycophenolic acid	HATU	**20** (20%)

## 3 Results and discussion

**Fig. 3:**
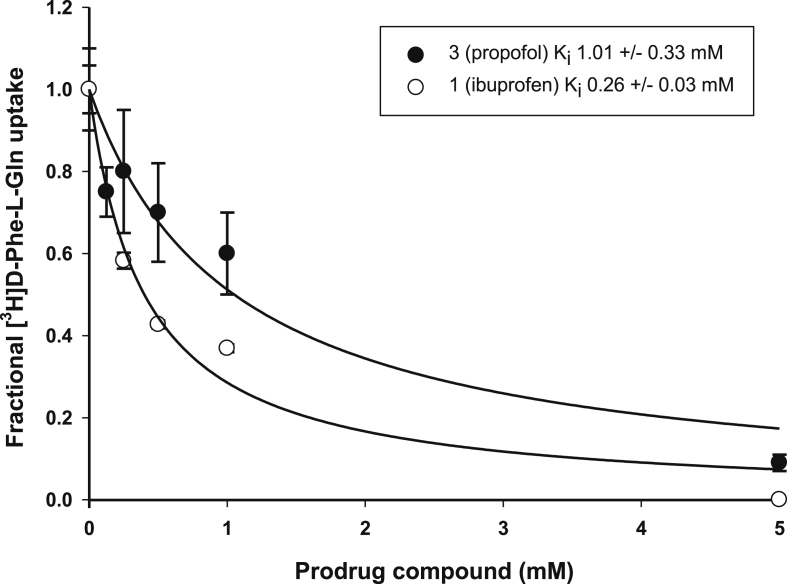
The K_i_ of prodrugs **1** and **3** for rabbit PepT1 expressed in *Xenopus laevis* oocytes.

The results of binding studies, *trans*-stimulation and Caco-2 monolayer assays are summarised in Table 2. The binding affinities of all prodrugs for PepT1 were determined by measuring the concentration at which they inhibit uptake of radiolabelled D-Phe-L-Gln in *Xenopus laevis* oocytes expressing rabbit PepT1. Inhibition constants were calculated from standard Michaelis-Menten kinetics [22,23]. PepT1 is a low affinity, high capacity transporter and compounds with an affinity <1 mM are generally classed as high affinity binders of the transporter. Fig. 3 shows the data for prodrugs **1** and **3**, which are representative of those determined for all the prodrugs. Prodrugs **4** and **5** had limited stability in the pH 5.5 assay buffer (multiple HPLC peaks), and so no reliable affinity or transport data could be generated.

**Table 2:** Summary of *in vitro* affinity and transport studies on rationally design prodrugs in oocytes and Caco-2 monolayers.

Compound (parent drug)	K_i_ (mM)	*Trans*-stimulated efflux?	Overall P_app_ (x 10^−6^ cm s^−1^) (Normalised^a^)	PepT1 P_app_ (x 10^−6^ cm s^−1^) (Normalised^a^)Ft
**FSA** (control)	0.32 ± 0.08	No	3.0 ± 0.5 (1.00)	1.9 ± 0.5 (1.00)
**1** (ibuprofen)	0.26 ± 0.03	Yes	3.7 ± 0.2 (1.29)	2.1 ± 0.3 (1.07)
**2** (gabapentin)	1.01 ± 0.33	Yes	0.6 ± 0.1 (0.46)	0.4 ± 0.1 (0.69)
**3** (propofol)	0.92 ± 0.19	Yes	0.5 ± 0.1 (0.41)	0.5 ± 0.1 (0.89)
**4** (aspirin)	nd^b^			
**5** (acyclovir)	nd^b^			
**6**^c^ (nabumetone)	0.08 ± 0.02	Yes	9.7 ± 0.1 (1.94)	4.4 ± 0.2 (1.30)
**7**^c^ (nabumetone)	0.46 ± 0.09	Yes	7.8 ± 0.2 (3.78)	6.3 ± 0.1 (6.52)
**8** (atenolol)	0.44 ± 0.15	No	nd	nd
**9** (zanamivir)	0.13 ± 0.02	No	nd	nd
**10** (baclofen)	1.87 ± 0.26	No	nd	nd
**11** (mycophenolate)	0.21 ± 0.08	Weak	nd	nd

a The PheΨ[CS-NH]-Ala (FSA) value is the mean ± RSD of six separate experiments with at least three monolayers. All other results are the mean ± RSD of one experiment with at least three monolayers. The normalised figure is to the FSA value recorded in that experiment.

b Prodrug was unstable to assay buffer (pH 5.5).

c Previously reported data [16].

**Fig. 4:**
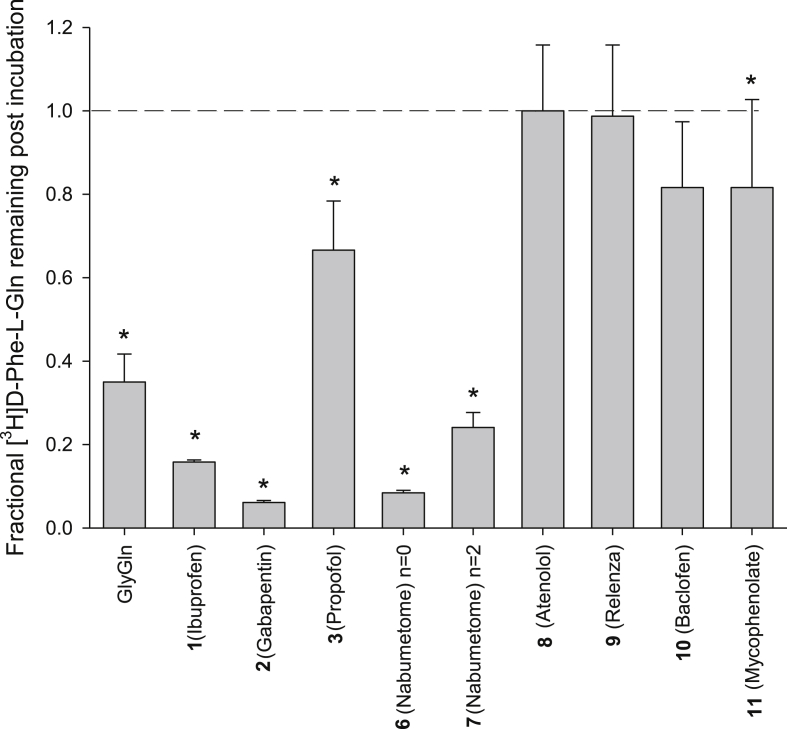
Effect of incubation of 10 mM prodrugs on the *trans*-stimulation efflux of radiolabelled [^3^H]-D-Phe-L-Gln, compared to the known PepT1 substrate GlyGln.

As binding studies only show affinity for PepT1 and do not provide information as to whether the compound is a substrate or an inhibitor, further transport experiments were undertaken. *Trans*-stimulation assays were performed using radiolabelled [^3^H]-D-Phe-L-Gln efflux from rabbit PepT1 expressing oocytes in the presence of 10 mM pro-drug. As controls, 10 mM Gly-L-Gln (a standard PepT1 substrate) or buffer lacking a substrate (negative control) were used. Fig. 4 shows the efflux data for the compounds summarised in Table 2.

Prodrugs **1**–**3**, **6**–**7** and **11** induced statistically significant *trans*-stimulation efflux in oocytes, thereby demonstrating PepT1 mediated transport [22,23]. Most of these induced similar or greater efflux than GlyGln, however prodrug **11** only weakly triggered efflux in comparison to GlyGln.

Prodrugs that generated robust *trans*-stimulated efflux in oocytes were further in a Caco-2 monolayer assay to investigate further the extent and rate of *trans*-epithelial transport. Caco-2 cells were chosen as they are widely accepted as a good overall model for the small intestinal epithelium [24], although it has been suggested that Caco-2 cells may underestimate the *in vivo trans*-epithelial rate of transport [25]. Apical to basolateral transport of 2 mM pro-drug, applied to the apical side, was monitored by high performance liquid chromatography (HPLC) after 1 h. The presence or absence of excess Gly-Gln allowed us to determine both the overall and PepT1 specific permeability (Table 2). The remaining pro-drugs were significantly transported in both oocyte and Caco-2 monolayer assays. The PepT1 mediated Papp values are of similar magnitude to known PepT1 (pro)drug substrates [10,16].

Based on these encouraging *in vitro* results, we elected to study **1** and **3** *in vivo* (Table 3), as simple examples of the two areas of interest to us. Administration of both **1** and **3** to rats resulted in intact prodrug being observed in the blood, with C_Max_ of 0.2 and 16.7 μg/mL respectively observed. Release of ibuprofen was also observed upon administration of **1**, with a relative bioavailability of 2% [26]. The shift in the T_Max_ observed for ibuprofen following administration of **1** when compared to free ibuprofen (from 1 h for free ibuprofen to 3.5 h for **1**) is indicative of a change in absorption mechanism. The low bioavailability of ibuprofen can be explained by the relative stability of the prodrug **1** to metabolism, as estimated by its *in vitro* half-life upon incubation with rat liver homogenate of over 17 h.

**Table 3:** Preliminary *in vivo* results. Values are mean ± standard deviation for n = 3 male Sprague-Dawley rats. IV = intra venous dosing.

Compound	Dose^a^ (mg kg^−1^)	Assayed compound	C_Max_ (μg mL^−1^)	AUC (μg h mL^−1^)	t_max_ (h)	Rat liver homgenate^b^ t_1/2_ (h)	Relative F (%)
Ibuprofen	6	Ibuprofen	5.7 ± 0.6	14.0 ± 1.8	0.7 ± 0.3		100 [26]
**1**	10	Intact **1**	0.2 ± 0.04	0.5 ± 0.1	1.0 ± 0.0	17.9	–
		Ibuprofen	0.2 ± 0.02	0.7 ± 0.1	3.2 ± 0.2		2.8 ± 0.3
**3**	7.6	Intact **3**	16.3 ± 1.8	3.2 ± 1.4	5.2 ± 0.8	7.3	–

a As free base.

b As compared to L-Trp-L-Ala, which had a t_1/2_ of 0.15 h under the same experimental conditions.

Regrettably, we were unable to detect free propofol released following administration of prodrug **3** because HPLC conditions to quantify free propofol could not be found. However, the relatively high C_Max_ for the intact prodrug **3** is encouraging and despite the relative metabolic stability of **3** (7-h half-life in rat liver homogenate) it is likely some free propofol would be available systemically. This is notable given the fact that propofol itself had no oral bioavailability in either rats or humans [27].

The low bioavailability of ibuprofen following administration of prodrug **1**, combined with fact that both prodrug **1** and **3** were relatively resistant to liver metabolism indicates that further work is required to design effective prodrugs suitable for therapeutic application. Nevertheless, we believe **1** and **3** serve as promising preliminary proof-of-concept to the idea that targeting PepT1 using our thiodipeptides can be used as a general strategy to overcoming oral bioavailability issues in drug discovery and development.

## 4 Conclusions

PepT1 is described in the literature as having a broad substrate capacity but, in reality, it has been limited to date to small peptides and peptide-like drugs. To better harness the capacity of this transporter as a drug delivery target, a rational and general targeting approach is required. We report here our data supporting a thiodipeptide prodrug as such a general targeting approach *in vitro* and *in vivo*.

We were excited to find that nine out of eleven of our rationally designed PepT1 targeting prodrugs displayed high affinity binding towards PepT1, and six of them triggered *trans*-stimulation. Additionally, prodrugs **1**–**3**, as well as **6**–**7** (as previously reported) [16], were all significantly transported in Caco-2 monolayers, with prodrugs **1** and **3** showing evidence of intact absorption *in vivo*. This provides proof-of-concept that diverse drug types can be delivered *via* a PepT1 mediated pathway using thiodipeptide carriers, with implications for future drug design strategies. Preliminary *in vivo* data also supports the use of thiodipeptide prodrugs to confer oral bioavailability.

The drugs exemplified represent examples of several classes of drugs for which oral delivery could be therapeutically interesting. These include thiodipeptide prodrugs of NSAIDs (e.g. **1**, **4**, **6**–**7**), which have high oral activity but also suffer from significant GI side effects. Examples of drugs for which oral activity is absent (e.g. **3**), low (e.g. **9**), or highly variable (**2**, **10**) have also been successfully modified using our thiodipeptide approach, to target the PepT1 transporter.

There is much future work to conduct before we can confidently say that rationally targeting PepT1 is a general strategy for oral drug delivery. In particular, the complete *in vivo* DMPK profile of our prodrugs needs to be established and future prodrugs need to be optimized for rapid liver and/or plasma esterase metabolism and release of free drug, or indeed potentially slower, sustained release if desired. Optimisation of the stability of the linker is also required, as evidenced by the instability of prodrugs **4** and **5**. However, our previously reported work suggests that the transporter can accommodate a wide variety of linkers [15,16], allowing scope to tailor the DMPK properties of a specific prodrug.

The oral delivery of drugs is a major challenge in pre-clinical development and leads to significant shelving of promising lead candidates in drug discovery. Our prodrug approach may allow many such biologically active compounds to be re-evaluated by administration as PepT1 targeting thiodipeptide prodrugs. Our *in vitro* and preliminary *in vivo* data is highly encouraging and warrants further work. In particular, our recent report that large peptide drugs such as cyclosporine A [28] can be rationally targeted towards PepT1 using the same approach offers the tantalising possibility of PepT1 targeting as a solution to the delivery of both small and peptidic molecules.

## 5 Experimental section

### 5.1 In vitro biological studies

The K_i_, *trans*-stimulation efflux and Caco-2 assays were performed as described previously [15,16].

Fresh rat liver homogenate was prepared by isolating liver from euthanized male rats, according to approved Home Office procedures. The liver was chopped with scissors and rinsed with medium (0.25 M sucrose, 25 mM KCl, 5 mM MgCl_2_ and 50 mM Tris/HCl, pH7.5) to remove trapped blood. The liver was then homogenized in fresh medium with a loose-fitting dounce-type homogenizer and kept on ice. Liver homogenate was incubated with either 0.5 mM compound **1** or **3**, or L-Trp-L-Ala as a positive control, at 37 °C. 250 μl aliquots of the homogenate were taken at 0, 0.25, 0.5, 1, 2, 6 and 24 h. The samples were precipitated by addition an equal volume of 3% perchloric acid and centrifugation at 17000*g* for 5 min. The perchloric acid was neutralized with 250 μl of 1 M KOH, and the sample subjected to a freeze/thaw cycle to precipitate the KClO_4_ salt before again being centrifuged. The supernatant was then analyzed by HPLC as for the Caco-2 permeability studies [15,16], and the half-life of the compounds calculated according to the method of Vig et al. [29].

### 5.2 In vivo studies

*In vivo* testing was carried out by Cyprotex Discovery Ltd., 15 Beech Lane, Macclesfield, Cheshire, SK10 2DR, United Kingdom or Saretius Ltd, Science & Technology Center, Earley Gate, University of Reading, Reading, Berkshire RG6 6BZ, United Kingdom.

Each test compound was administered orally as solutions in distilled water (ibuprofen, **1**) or polypropylene glycol (**3**) to three adult male Sprague-Dawley weighing 250–300 g, which were housed singly following jugular vein cannulation prior to administration of compound. Animals were given free access to food and water throughout the study and maintained under a 12-h light/dark cycle with temperature and humidity controlled according to Home Office regulations. All compounds were well-tolerated and no-adverse events were reported.

Blood samples (230 μL) were taken from the carotid artery at the following time points and placed in heparinized tubes: predose, 0.25, 0.5, 1, 2, 4, 8 and 24 h post dose. After the final time point the animals were sacrificed by an overdose of anaesthetic.

Blood samples were centrifuged to obtain the plasma, which was transferred to a separate labelled container. Aliquots from the individual time points for the three animals were analyzed singly. 80 μL of plasma was diluted with 20 μL of 1:1 ACN:water, then 800 μL chilled ACN was added, samples briefly vortex mixed and the centrifuged at 13000 rpm for 5 min at 4 °C. 500 μL of the resultant supernatant was further diluted with 500 μL water.

20 μL sample was analyzed by LC-MS/MS using a C18 5 μm Gemini UHPLC column running a gradient of 90% 0.04% acetic acid in water to 90% ACN over 3 min at a flow rate of 0.5 mL min^−1^. MS data was acquired under multiple reaction monitoring conditions using a turbo spray ion source. The concentration in the plasma was determined by comparison to standard curves of the administered compound prepared in blank plasma matrices and treated in an identical manner to the samples.

### 5.3 Synthetic chemistry

Anhydrous solvents and reagents were obtained as follows: DMF was dried three times over molecular sieves (3 Å). THF was dried by distillation from sodium benzophenone ketyl, DCM and toluene by distillation from calcium hydride. All reactions were conducted at room temperature in dry glassware under a nitrogen atmosphere, unless otherwise stated. All chemicals were used directly from suppliers' (Sigma-Aldrich) vessel without further purification, unless otherwise stated. Protected amino acids and HBTU were supplied by Novabiochem. ^1^H NMR spectra were recorded at 300, 400 or 500 MHz and ^13^C NMR spectra at 75, 100 or 125 MHz on a Bruker AC300, AC400, Avance II or Varian Unity INOVA 300 spectrometers. Chemical shifts are denoted in ppm (*δ*) relative to the internal solvent standard. The splitting patterns for NMR spectra are designated as follows: s (singlet), br (broad), d (doublet), t (triplet), p (pentet), m (multiplet), or combinations thereof. Coupling constants (*J*) are designated in Hz and reported to 1 decimal place. Assignments were made with the aid of DEPT135, COSY and HMQC experiments. ES-MS (and HRMS) spectra were recorded on a Micromass LCT orthogonal acceleration time-of-flight mass spectrometer (positive ion mode) with flow injection *via* a Waters 2790 separation module autosampler. IR spectra were obtained using a Nicolet-Nexus 670/680 F  T-IR or ATI Mattson Genesis Series FT-IR spectrometer and are quoted in cm^−1^. Optical rotations were measured at 589 nm in a 1 dm cell using an Optical Activity AA1000 polarimeter and are quoted in 10^−2^ deg cm^2^ g^−1^. Melting point determinations were made using a Stuart Scientific SMP1 apparatus and are uncorrected. Analytical TLC was performed on Merck silica gel 60 F_254_ aluminium backed plates. The plates were visualised under UV fluorescence (254 nm) or developed using ninhydrin (0.5% w/v butanol), 2-bromocresol or acidified potassium permanganate solution with charring as necessary. R_f_ values are reported to the nearest 0.01. Mixed solvent system compositions are quoted as volumetric ratios. Column chromatography employed BDH silica gel (50–70 μm). Reverse phase analytical HPLC was performed on a Grace ODS (4.6 × 150 mm) column using a Gilson 306 pump with a flow rate of 1 mL min^−1^. Detection was at 254 nm by a Gilson 115 UV detector. Reverse phase semi-preparative HPLC was performed on identical apparatus using a Varian ODS 10u (21.2 × 250 mm) column and a 15 mL min^−1^ flow rate. DataApex Clarity™ software was used for integration and analysis. Retention times (in minutes) are quoted to one decimal place for the analytical system and are followed in parenthesis by the solvent conditions (v/v) used. Silica analytical and semi-preparative HPLC was performed using identical apparatus, software and respective flow rates with Silica PhenoSphere™ (4.6 × 250 mm) and Rainin Dynamax™ 60 A (21.4 × 250 mm) columns respectively. Retention times (in minutes) are quoted to one decimal place for the analytical system and are followed in parenthesis by the solvent conditions (v/v) used.

#### 5.3.1 (S)-3-{2-[1-(*tert*-Butoxycarbonylamino-methyl)-cyclohexyl]-acetoxy}-2-((S)-2-*tert*-butoxycarbonylamino-thiopropionylamino)-propionic acid *tert*-butyl ester (**14**)

Gabapentin (85 mg; 0.5 mmol) and di-*tert*-butyl dicarbonate (130 mg; 0.6 mmol) were suspended in 1 mL DMF. TEA (0.07 mL; 0.5 mmol) was added and the suspension stirred for four days at room temperature. A solution was formed after the first 3 h of stirring. The DMF was removed *in vacuo*. HBTU (245 mg; 0.6 mmol) and DIPEA (0.11 mL; 0.6 mmol) were added to the residue, which was then redissolved in 1 mL DMF. The resultant solution was stirred at room temperature for 30 min **12** (132 mg; 0.4 mmol) in 1 mL DMF was then added and the solution stirred for three days at room temperature. The DMF was removed and the residue purified by flash column chromatography (4:1 hexane:EtOAc → 1:1 hexane:EtOAc), followed by semi-preparative HPLC (2:1 hexane:EtOAc) to give title compound as a colourless oil (52 mg; 22%). HPLC R_T_: 4.2 (2:1 hexane:EtOAc). [α]_D_^31^ (CHCl_3_; c = 3.68): −20.20. υ_max_ (thin film): 3344, 2979, 2931, 2864, 1694, 1514, 1455, 1393, 1367, 1250, 1163, 1102, 1049, 994, 913, 847, 733. *δ*_H_ (500 MHz, CDCl_3_): 1.51–1.26 (40H, m), 2.31–2.22 (2H, m), 3.07 (1H, dd, *J* = 14.0 & 6.5), 3.17 (1H, dd, *J* = 14.0 & 6.5), 4.62–4.40 (3H, m), 4.91 (1H, s), 5.25 (1H, s), 5.50 (1H, s), 8.99 (1H, s). *δ*_C_ (125 MHz, CDCl_3_): 21.2, 21.8*, 25.7, 27.7, 27.8*, 28.2*, 28.2, 28.3*, 28.3, 33.6, 33.8, 37.4, 39.8, 46.9, 56.1, 57.4, 62.7, 79.2, 79.7, 83.1, 154.9, 156.3, 156.9*, 166.9, 171.6, 206.3, 208.8*. HRMS: Calculated C_29_H_52_N_3_O_8_S: 624.3289, found: 624.3280. * minor rotamer.

#### 5.3.2 (S)-3-[2-(1-Aminomethyl-cyclohexyl)-acetoxy]-2-((S)-2-amino-thiopropionylamino)-propionic acid (**2**)

Deprotection of 44 mg of **14** as for compound **1**. Yield of **2** as di-TFA salt = 40 mg; 97%. HPLC R_T_: 4.6 (10%, Chromolith^®^). *δ*_H_ (500 MHz, CD_3_OD): 1.36–1.45 (3H, m), 1.47–1.59 (10H, m), 2.57 (2H, q, *J* = 15.5), 3.06 (2H, s), 4.35 (1H, q, *J* = 6.5), 4.53 (1H, dd, *J* = 11.5 & 5.5), 4.62 (1H, dd, *J* = 11.5 & 3.5), 5.30 (0.1H, t, *J* = 5.0)*, 5.47 (0.9H, t, *J* = 5.0). *δ*_C_ (100 MHz, CD_3_OD): 19.5*, 19.7, 20.9*, 21.1, 22.7*, 25.4*, 25.6, 25.7*, 32.9*, 33.0, 33.1*, 35.2, 36.5, 47.2, 53.8, 54.5, 63.1*, 63.3, 171.6, 201.5. MS: C_15_H_27_N_3_O_4_S *m*/*z* (ES^−^) 361.4 [M-H^+^+NH_3_]. * minor rotamer.

#### 5.3.3 (S)-2-((S)-2-*tert*-Butoxycarbonylamino-thiopropionylamino)-succinic acid 1-*tert*-butyl ester 4-(2,6-diisopropyl-phenyl) ester (**15**)

**13** (120 mg; 0.3 mmol) and DCC (83 mg; 0.4 mmol) were stirred in 1 mL DMF for 1 h at 0 °C. A precipitate was formed during this time. DMAP (7 mg; 0.1 mmol) and 2,6-diisopropylphenol (0.07 mL; 0.4 mmol) were added. The suspension turned yellow immediately, was warmed to room temperature and stirred for 24 h. The mixture was filtered and the residue washed with DCM. The solvent was removed *in vacuo* and the residue purified by flash column chromatography (DCM → 9:1 DCM:Et_2_O) to give title compound as a yellow oil (66 mg; 38%). R_f_ (95:5 DCM:Et_2_O): 0.58. [α]_D_^31^ (CHCl_3_; c = 3.00): +60.20. υ_max_ (thin film): 3323, 2980, 2934, 1728, 1514, 1396, 1370, 1335, 1248, 1158, 1053, 912, 846, 734. *δ*_H_ (500 MHz, CDCl_3_): 1.18 (12H, d, *J* = 5.5), 1.50–1.41 (21H, m), 2.95–2.70 (2H, m), 3.44–3.35 (1H, m), 3.66–3.55 (1H, m), 4.49 (1H, t, *J* = 6.5), 5.13 (1H, s), 5.28 (1H, s), 7.15 (2H, d, *J* = 7.5), 7.22 (1H, t, *J* = 7.5), 8.56 (0.2H, br s)*, 8.69 (0.8H, d, *J* = 7.0). *δ*_C_ (100 MHz, CDCl_3_): 22.1, 22.8, 24.4, 27.8, 28.2, 34.2, 53.7, 53.9, 83.4, 124.0, 126.8, 140.0, 145.2, 155.1, 168.1, 170.3, 204.9.

#### 5.3.4 (S)-2-((S)-2-Amino-thiopropionylamino)-succinic acid 4-(2,6-diisopropyl-phenyl) ester (**3**)

Deprotection of 66 mg of **15** as for compound **1**. Yield of **3** as TFA salt = 23 mg; 39%. HPLC R_T_: 4.8 (50%, Chromolith^®^). *δ*_H_ (500 MHz, CD_3_OD): 1.17 (6H, s), 1.18 (6H, s), 1.49 (0.8H, dd, *J* = 7.5 & 6.5)*, 1.49 (2.2H, d, *J* = 6.5), 2.85–3.05 (2H, m), 3.22–3.55 (2H, m), 4.27 (0.8H, q, *J* = 7.0), 4.33 (0.2, q, *J* = 7.0)*, 5.53 (1H, br s), 7.15–7.23 (3H, m). *δ*_C_ (100 MHz, CD_3_OD): 20.9, 23.5, 24.2, 28.3, 28.7, 35.7, 55.2, 55.7, 125.1, 127.9, 141.8, 146.8, 171.1, 171.9, 202.3. MS: C_19_H_28_N_2_O_4_S *m*/*z* (ES^+^) 381.1 [M + H^+^]. HRMS: Calculated C_19_H_29_N_2_O_4_S: 381.1843, found: 381.1836. * minor rotamer.

#### 5.3.5 2-(2-Oxo-propyl)-benzoic acid 2-[2-(2-hydroxy-ethoxy)-ethoxy]-ethyl ester (**21**)

A suspension of aspirin (345 mg; 1.9 mmol), triphenylphosphine (545 mg; 2.1 mmol) and triethylene glycol (0.27 mL; 2.0 mmol) in 0.7 mL THF was sonicated to give a viscous solution. DIAD (0.40 mL; 2.0 mmol) was then added over 5 min to give a yellow solution. This was sonicated for 15 min at room temperature. The solution was purified by flash column chromatography (4:1 hexane:EtOAc → EtOAc) to give crude product. This was further purified by flash column chromatography (9:1 DCM:Et_2_O → EtOAc) to give the title compound as a colourless oil (294 mg; 49%). R_f_ (EtOAc): 0.29. υ_max_ (thin film): 3489, 2875, 1768, 1723, 1608, 1486, 1453, 1369, 1295, 1258, 1197, 1123, 1082, 916, 754. *δ*_H_ (500 MHz, CDCl_3_): 2.35 (3H, s), 2.63 (1H, br s), 3.58 (2H, t, *J* = 4.4), 3.66 (4H, br s), 3.68–3.70 (2H, m), 3.78 (2H, t, *J* = 4.7), 4.43 (2H, t, *J* = 4.7), 7.09 (1H, d, *J* = 8.2), 7.30 (1H, t, *J* = 7.6), 7.55 (1H, t, *J* = 8.2), 8.05 (1H, d, *J* = 7.9). *δ*_C_ (150 MHz, CDCl_3_): 20.9, 61.6, 64.0, 69.0, 70.2, 70.6, 72.4, 123.0, 123.7, 125.9, 131.8, 133.9, 150.6, 164.4, 169.7. MS: C_15_H_20_O_7_ *m*/*z* (ES^+^) 335.1 [M + Na^+^]. HRMS: Calculated C_15_H_20_O_7_Na: 335.1101, found: 335.1092.

#### 5.3.6 (S)-2-((S)-2-*tert*-Butoxycarbonylamino-thiopropionylamino)-succinic acid 4-(2-{2-[2-(2-acetoxy-benzoyloxy)-ethoxy]-ethoxy}-ethyl) ester 1-*tert*-butyl ester (**22**)

**13** (142 mg; 0.4 mmol) and HBTU (294 mg; 0.8 mmol) was dissolved in 2 mL DMF. DIPEA (0.08 mL; 0.5 mmol) was added to the colourless solution which turned orange almost immediately. The solution was stirred at room temperature for 1 h at which time it was red/brown in colour. **21** (287 mg; 0.9 mmol) in 1 mL DMF was then added and the solution stirred for four days at room temperature. The DMF was removed under vacuum and the residue purified by flash column chromatography (1:1 hexane: EtOAc → EtOAc) to give the title compound as a yellow oil (202 mg; 80%). R_f_ (1:1 hexane:EtOAc): 0.26. [α]_D_^20^ (CHCl_3_; c = 1.14): +27.94. υ_max_ (thin film): 3337, 2978, 2934, 1762, 1723, 1607, 1513, 1453, 1393, 1254, 1195, 1083, 1047, 1012, 916, 848. *δ*_H_ (500 MHz, CDCl_3_): 1.39–1.42 (21H, m), 2.32 (3H, s), 3.00 (1H, dd, *J* = 17.1 & 3.9), 3.11 (1H, dd, *J* = 17.1 & 4.0), 3.62–3.67 (6H, m), 3.75–3.77 (2H, m), 4.12–4.23 (2H, m), 4.40 (2H, t, *J* = 4.7), 4.42–4.48 (1H, m), 5.17–5.18 (0.8H), 5.24–5.25 (0.2H, m)*, 5.31–5.33 (0.2H)*, 5.44 (0.8H), 7.07 (1H, dd, *J* = 7.9 & 1.1), 7.27 (1H, dd, *J* = 7.9, 7.6 & 1.1), 7.53 (1H, dd, *J* = 7.9, 7.6 & 1.6), 8.01 (1H, dd, *J* = 7.9 & 1.6), 8.66 (0.8H, d, *J* = 7.5), 8.73 (0.2H, d, *J* = 7.5)*. *δ*_C_ (150 MHz, CDCl_3_): 20.8, 21.8, 27.7, 28.1, 34.7, 53.9, 56.8, 63.8 & 69.0, 68.9, 70.2, 70.4, 79.9, 82.9, 122.9, 123.7, 125.8, 131.7, 133.8, 150.5, 154.7, 164.2, 168.2, 169.5, 170.3, 170.8*, 205.0. MS: C_31_H_46_N_2_O_12_ S *m*/*z* (ES^+^) 688.6 [M + NH_4_^+^]. HRMS: Calculated C_31_H_50_N_3_O_12_S: 688.3110, found: 688.3125. * minor rotamer.

#### 5.3.7 (S)-2-((S)-2-Amino-thiopropionylamino)-succinic acid 4-(2-{2-[2-(2-acetoxy-benzoyloxy)-ethoxy]-ethoxy}-ethyl) ester (**4**)

Deprotection of 65 mg of **22** as for compound **1**. Yield of **4** as TFA salt = 52 mg; 85%. R_f_ (1:1:1:1 EtOAc:BuOH:H_2_O:CH_3_CO_2_H): 0.42. [α]_D_^24^ (MeOH; c = 0.82): +29.44. *δ*_H_ (500 MHz, D_2_O): 1.40 (0.4H, d, *J* = 6.9)*, 1.46 (2.6H, d, *J* = 6.9), 2.27 (3H, s), 2.87–3.01 (2H, m), 3.52–3.68 (6H, m), 3.72–3.78 (1.7H, m), 3.79–3.82 (0.3H, m)*, 4.00–4.07 (0.3H, m)*, 4.08–4.15 (1.7H, m), 4.33 (1H, q, *J* = 6.9), 4.31–4.38 (1.7H, m), 4.37–4.33 (0.3H, m)*, 5.15–5.28 (1H, m), 7.12 (1H, dd, *J* = 7.9 & 1.3), 7.34 (1H, dd, *J* = 7.9, 7.6 & 1.3), 7.60 (1H, dd, *J* = 7.9, 7.6 & 1.6), 7.94 (1H, dd, *J* = 7.9 & 1.6). *δ*_C_ (150 MHz, D_2_O): 17.7*, 18.2, 18.6, 38.3, 50.9, 51.1, 62.1, 65.9, 67.2, 67.9, 69.3, 120.1, 121.7*, 124.5, 129.4, 131.4, 137.4, 147.2, 160.5, 163.8, 169.9, 170.9, 198.7. MS: C_22_H_30_N_2_O_10_ S *m*/*z* (ES^+^) 515.4 [M + H^+^]. HRMS: Calculated C_22_H_31_N_2_O_10_S: 515.1694, found: 515.1697. * minor rotamer.

#### 5.3.8 (S)-2-((S)-2-*tert*-Butoxycarbonylamino-thiopropionylamino)-succinic acid 4-[2-(2-amino-6-oxo-1,6-dihydro-purin-9-ylmethoxy)-ethyl] ester 1-*tert*-butyl ester (**16**)

**13** (124 mg; 0.3 mmol) and DCC (68 mg; 0.3 mmol) were dissolved in 0.3 mL DMF and cooled to 0 °C. The reaction was stirred at this temperature for 1 h during which time a white precipitate was formed. A solution of acyclovir (50 mg; 0.2 mmol) and DMAP (10 mg; 0.1 mmol) in 1.5 mL DMF was then added. The suspension was warmed to room temperature and stirred for 24 h during which time the suspension changed from orange-brown to a brown-purple colour. The suspension was filtered and the filtrate washed with the minimum of DCM. The liquor was concentrated and the residue purified by flash column chromatography (95:5 CHCl_3_:MeOH → 9:1 CHCl_3_:MeOH) to give the title compound as a brown solid (123 mg; 96%). R_f_ (CHCl_3_:CH_3_OH, 9:1): 0.46. [α]_D_^25^ (CHCl_3_; c = 0.98): +7.20. υ_max_ (thin film): 3311, 3107, 2982, 2941, 1696, 1602, 1534, 1484, 1371, 1254, 1157, 1102, 1057, 845, 758. *δ*_H_ (CD_3_OD, 300 MHz): 1.41 (3H, d, *J* = 6.8), 1.49–1.63 (18H, m), 2.88–3.03 (2H, m), 3.71–3.80 (2H, m), 4.14–4.22 (2H, m), 4.41–4.48 (1H, m), 5.23–5.28 (1H, m), 5.49 (2H, s), 7.88 (1H, s). *δ*_C_ (CD_3_OD, 75 MHz): 22.2, 28.5, 29.1, 36.1, 56.2, 57.5, 65.2,65.2, 68.7, 68.7, 74.1, 79.9, 84.2, 118.7, 140.2, 153.2, 156.0, 156.9, 159.6, 170.3, 172.0, 206.5. MS: C_24_H_38_N_7_O_8_S *m*/*z* (ES^+^) 584.1 [M + Na^+^]. HRMS: Calculated C_24_H_38_N_7_O_8_SNa: 584.2497, found: 584.2503.

#### 5.3.9 (S)-2-((S)-2-Amino-thiopropionylamino)-succinic acid 4-[2-(2-amino-6-oxo-1,6-dihydro-purin-9-ylmethoxy)-ethyl] ester (**5**)

A mixture of **16** (51 mg; 0.1 mmol) and phenol (179 mg; 1.9 mmol) was heated to 45 °C at which point the now liquid phenol had dissolved. Trifluoroacetic acid (0.03 mL; 0.4 mmol) was then added and the solution stirred at 45 °C for 1 h. The solution was diluted with 2 mL EtOAc and washed three times with 3 mL water. The EtOAc was removed *in vacuo* and the residue sequentially taken up in DMF and water, both of which were removed under vacuum. The resultant residue, which was free from phenol contamination, was redissolved in 5 mL water and lyophilised to give di-TFA salt of **5** as an off white solid (28 mg; 44%). *δ*_H_ (D_2_O, 300 MHz): 1.37 (0.4H, d, *J* = 7.0)*, 1.50 (2.6H, d, *J* = 7.0), 2.80–2.91 (2H, m), 3.77–3.84 (2H, m), 4.13–4.20 (2H, m), 4.26 (1H, q, *J* = 6.9), 5.03–5.11 (1H, m), 5.47 (2H, s), 8.09 (0.9H, s), 8.50 (0.1H, s)*. *δ*_C_ (D_2_O, 75 MHz): 19.8, 34.7, 53.9, 54.2, 54.4, 54.8*, 64.2, 67.8, 74.3, 118.4, 138.5, 150.6, 162.9, 171.8, 172.4, 201.2. MS: C_15_H_21_N_7_O_6_S *m*/*z* (ES^+^) 428.0 [M + H^+^]. HRMS: Calculated C_15_H_22_N_7_O_6_S: 428.1347, found: 428.1336. * minor rotamer.

#### 5.3.10 (*S*)-4-((*S*)-3-(4-(2-amino-2-oxoethyl)phenoxy)-1-(*tert*-butoxycarbonyl)propan-2-yl) 1-*tert*-butyl 2-((*S*)-2-(*tert*-butoxycarbonyl)propanethioamido)succinate (**17**)

*S* (−)-Atenolol (100 mg, 0.37 mmol) was dissolved in water (10 ml) containing (59 mg, 0.56 mmol) of sodium carbonate and the mixture was cooled to 0 °C with stirring. Then, the solution of di-*tert*. butyl dicarbonate (125 mg, 0.57 mmol) in 10 ml of 1,4-dioxane was slowly added at the same temperature. The mixture was stirred overnight at room temperature, evaporated in *vacuo* to get white solid, diluted with 20 ml of water, and extracted with three portions of ethyl acetate. The ethyl acetate solution was washed with brine, dried (MgSO_4_) and evaporated in *vacuo* to afford white solid (131 mg). A solution of this in 10 mL MeCN was slowly added over 20 min to a solution of **13** (150 mg, 0.39 mmol) in MeCN (15 ml) containing (117 mg, 0.71 mmol) of 1,1′-carbonyl diimidazole and (14 mg, 0.2 mmol) of imidazole. The mixture was stirred for 3 days at room temperature under the inert atmosphere. The precipitated solid was filtered off and the filtrate was evaporated in *vacuo* to get thick brown gel, which can further diluted with 30 ml of EtOAc, and washed with 2 M HCl (2 × 10 ml), saturated aqueous NaHCO_3_ (3 × 10 ml), water (2 × 10 ml) and finally with the brine (20 ml), dried (MgSO_4_) and evaporated in *vacuo* to afford white crude solid. The residue was purified by flash column chromatography (3:7 petrol:EtOAc → EtOAc) to give desired product as a white solid (130 mg; 36%). M. p. 64–67 °C. R_*f*_ 0.40 [Petrol-EtOAc 2:8]. [α]_D_^25^ (CHCl_3_; c = 0.028): 34.97. υ_max_ (neat)/cm^−1^ 3675, 3338, 2973, 2901, 1690, 1667, 1614, 1511, 1453, 1406, 1393, 1366, 1242, 1155, 1050, 900, 846, 775, 731, 435. *δ*_H_ (300 MHz, CDCl_3_): 1.10 (6H, s), 1.32–1.42 (30H, m), 2.91 (1H, br s), 3.35 (1H, br s), 3.40–3.45 (3H, m), 3.92–4.12 (3H, m), 4.33–4.45 (1H, m), 5.10–5.22 (1H, m), 5.22–5.30 (1H, m), 5.30–5.42 (1H, m), 5.70–5.80 (1H, m), 6.82 (2H, d, *J* = 8.7), 7.15 (2H, d, *J* = 8.5). *δ*_C_ (75 MHz, CDCl_3_): 17.5, 21.0, 22.1, 22.6, 23.8, 27.8, 28.2, 28.4, 29.3, 29.6, 31.9, 34.9, 42.3, 43.2, 53.9, 56.0, 57.0, 67.4, 80.1, 83.1, 115.1, 127.5, 130.6, 154.8, 157.6, 168.2, 174.1, 205.4. HRMS: Calculated C_35_H_56_N_4_O_10_S 725.3790, found 725.3795. MS C_35_H_55_N_4_O_10_ S *m*/*z* (ES^+^) 725.37 [M + H^+^].

#### 5.3.11 (*S*)-4-((*S*)-3-(4-(2-amino-2-oxoethyl)phenoxy)-1-(isopropylamino)propan-2-yloxy)-2-((*S*)-2-aminopropanethioamido)-4-oxobutanoic acid (**8**)

**17** (100 mg, 0.138 mmol) was dissolved in 3 mL 97% formic acid. The solution was refluxed at 100 °C for 3 h, followed by room temperature for overnight. The excess formic acid was then removed under high vacuum and the residue taken up in 2 mL distilled water. The fine suspension was filtered through a pipette plugged with glass wool and lyophilised to give the formate salt of **8** as a brown solid (53 mg; 76%). M. p. 118–121 °C [α]_D_^29^ (MeOH; c = 0.028): −35.71. υ_max_ (neat)/cm^−1^ 3648, 3195, 2972, 2116, 1869, 1830, 1738, 1667, 1583, 1510, 1456, 1380, 1346, 1298, 1240, 1176, 1082, 1066, 1046, 920, 879, 798, 763, 668, 567, 518. *δ*_H_ (300 MHz, D_2_O): 1.1 (6H, d, *J* = 6.4), 1.42 (3H, d, *J* = 6.8), 2.73–2.92 (2H, m), 3.22–3.41 (5H, m), 4.10–4.25 (3H, m), 4.91 (1H, t, *J* = 6.4), 5.31–5.42 (1H, m), 6.83 (2H, d, *J* = 8.7), 7.15 (2H, d, *J* = 8.7), 8.23 (2H, s). *δ*_C_ (75 MHz, D_2_O): 17.7, 18.1, 19.2, 35.4, 40.6, 44.7, 51.4, 53.7, 56.7, 66.9, 68.9, 114.9, 128.2, 130.5, 156.6, 167.8, 174.7, 177.7, 199.3. MS: C_21_H_32_N_4_O_6_S *m*/*z* (ES^+^) 468.2 [M^+^]. HRMS: Calculated for C_21_H_32_N_4_O_6_S 468.2043, found 468.1999.

#### 5.3.12 (4*S*,5R,6R)-5-acetamido-6-((1R,2R)-3-((*S*)-4-*tert*-butoxy-3-((*S*)-2-(*tert*-butoxycarbonyl)propanethioamido)-4-oxobutanoyloxy)-1,2-dihydroxypropyl)-4-guanidino-5,6-dihydro-4*H*-pyran-2-carboxylic acid (**18**)

A mixture of **13** (100 mg, 0.26 mmol), HATU (117 mg, 0.30 mmol) and DIPEA (0.15 ml, 0.8 mmol) in anhydrous DMF (10 ml) was stirred under nitrogen at 0 °C for 30 min then a solution of Relenza (97 mg, 0.29 mmol) in the mixture of dry DMF:DMSO(10 ml, 8:2) was added and stirring was continued for another 3 days at room temperature. The reaction mixture were filtered off and the filtrate, plus a DMF washing, was evaporated in *vacuo* to get crude oil, which was further purified by chromatography, eluting with neat EtOAC to 1:1 (MeOH: EtOAC) to give desired product as an off white solid (20 mg, 11%). M. p. 282–284 °C. R_*f*_ 0.40 [MeOH-EtOAc 3:7]. [α]_D_^25^ (CH_3_OH; c = 0.028): 35.71. υ_max_ (neat)/cm^−1^ 3668, 3244, 2988, 2972, 2901, 1704, 1689, 1568, 1453,1405, 1322, 1250, 1155, 1049, 894, 609, 548. *δ*_H_ (300 MHz, CD_3_OD): 1.40 (22H, s), 2.00 (5H, s), 2.65 (1H, s), 3.00–3.10 (2H, m), 3.40 (1H, br s, -O**H**), 3.60–3.75 (1H, m, 3.75–3.90 (1H, m), 4.00–4.25 (4H, m), 4.30–4.50 (3H, m), 5.20–5.25 (1H, m), 5.50 (1H, s). *δ*_C_ (75 MHz, D_2_O): 21.8, 26.9, 27.4, 47.6, 48.0, 51.0, 62.9, 67.9, 69.6, 75.2, 82.0, 103.8, 130.0, 149.0, 152.0, 156.8, 170.0, 174.2, 184.0. MS: C_28_H_46_N_6_O_12_ S *m*/*z* (ES^+^) 691.3 [M + H^+^]. HRMS Calculated for C_28_H_47_N_6_O_12_S 691.2967, found 691.2973.

#### 5.3.13 (4*S*,5R,6R)-5-acetamido-6-((1R,2R)-3-((*S*)-3-((*S*)-2-aminopropanethioamido)-3-carboxypropanoyloxy)-1,2-dihydroxypropyl)-4-guanidino-5,6-dihydro-4*H*-pyran-2-carboxylic acid (**9**)

Deprotection of 100 mg of **18** as for compound **8**. Yield of **9** as formate salt = 54 mg; 70%. M. p. 210–212 °C. [α]_D_^29^ (H_2_O; c = 0.057): 17.54. υ_max_ (neat)/cm^−1^ 3325, 3178, 2924, 2111, 1717, 1680, 1589, 1374, 1324, 1282, 1146, 1041, 945, 768, 665. *δ*_H_ (300 MHz, D_2_O): 1.42 (3H, s), 1.92 (3H, s), 2.73–2.90 (3H, m), 3.45–3.55 (2H, m), 3.73–3.82 (2H, m), 4.02–4.11 (1H, m), 4.15–4.30 (3H, m), 4.82–4.91 (1H, m), 5.53 (1H, s), 8.22 (1H, s). *δ*_C_ (75 MHz, D_2_O): 19.2, 21.8, 37.0, 47.5, 50.9, 53.0, 57.2, 62.0, 67.9, 69.6, 75.2, 104.1, 136.0, 150.0, 156.8, 174.2, 180.0, 199.6. MS: C_19_H_30_N_6_O_10_ S *m*/*z* (ES^+^) 534.2 [M^+^]. HRMS: Calculated for C_19_H_30_N_6_O_10_S 534.1744, found 534.1698.

#### 5.3.14 (*R*)-((*S*)-3-*tert*-butoxy-2-((*S*)-2-(*tert*-butoxycarbonyl)propanethioamido)-3-oxopropyl) 4-(*tert*-butoxycarbonyl)-3-(4-chlorophenyl)butanoate (**19**)

A mixture of Boc-baclofen (210 mg, 0.66 mmol), DCC (140.5 mg, 0.68 mmol) and DMAP (5 mg, 0.05 mmol) in anhydrous DCM (20 ml) was stirred at room temperature for 15 min under the inert atmosphere. Then, the solution of **12** (163 mg, 0.468 mmol) in 10 ml of DCM was slowly added at the same temperature. The mixture was stirred for 5 h at room temperature under the inert atmosphere. The precipitated solid was filtered off and the filtrate was evaporated in *vacuo* to get viscous liquid, which can further diluted with 30 ml of EtOAc, and washed with 2 M HCl (2 × 10 ml), saturated aqueous NaHCO_3_ (3 × 10 ml), water (2 × 10 ml) and finally with the brine (20 ml), dried (MgSO_4_) and evaporated in *vacuo* to afford white crude solid. The residue was purified by flash column chromatography (9:1 petrol:EtOAc → 7:3 petrol:EtOAc) to give desired product as a white solid (280 mg; 65%). M. p. 65–68 °C, R_*f*_ 0.50 [Petrol-EtOAc 8:2]. [α]_D_^28^ (CHCl_3_; c = 0.028): 71.43. υ_max_ (neat)/cm^−1^: 3325, 2976, 2928, 2851, 1991, 1693, 1626, 1574, 1510, 1493, 1449, 1411, 1393, 1366, 1310, 1244, 1190, 1108, 1088, 1048, 1014, 967, 843, 827, 641, 534. *δ*_H_ (300 MHz, CDCl_3_): 1.42 (30H, s), 2.53 (1H, dd, *J* = 7.5 & 6.4), 2.62 (1H, dd, *J* = 7.5 & 6.4) 3.11–3.33 (2H, m), 3.33–3.42 (1H, m), 4.35 (1H, dd, *J* = 11.5 & 2.8), 4.41–4.53 (2H, m), 4.55 (1H, dd, *J* = 11.5 & 2.4), 5.15–5.25 (1H, br m), 5.62 (1H, br s), 7.15 (2H, d, *J* = 8.5), 7.25 (2H, d, *J* = 8.3), 8.83 (1H, br s). *δ*_C_ (75 MHz, CDCl_3_): 21.7, 27.8, 28.3, 28.38, 33.8, 37.6, 41.3, 57.1, 57.3, 62.9, 79.4, 82.7, 83.4, 128.9, 132.9, 139.7, 155.9, 167.2, 170.9, 206.2. MS: C_30_H_46_ClN_3_O_8_S *m*/*z* (ES^+^) 644.3 [M + H^+^]. HRMS: Calculated for C_30_H_47_ClN_3_O_8_S 644.2195, found 644.2759.

#### 5.3.15 (*S*)-3-((*R*)-4-amino-3-(4-chlorophenyl)butanoyloxy)-2-((*S*)-2- aminopropanethioamido)propanoic acid (**10**)

Deprotection of 100 mg of **19** as for compound **8**. Yield of **10** as formate salt = 54 mg; 80%. M. p. 112–115 °C [α]_D_^28^ (H_2_O; c = 0.057): −17.54. υ_max_ (neat)/cm^−1^: 3195, 2979, 2112, 1869, 1730, 1704, 1688, 1582, 1511, 1490, 1456, 1379, 1241, 1164, 1110, 1089, 1050, 1013, 897, 823, 764, 668, 527; *δ*_H_ (300 MHz, D_2_O): 1.42 (3H, s), 2.45–2.62 (1H, m), 2.62–2.75 (1H, m), 3.05–3.15 (1H, m), 3.15–3.35 (2H, m), 4.25–4.35 (1H, m), 4.35–4.45 (1H, m), 4.82–5.01 (2H, m), 7.22 (2H, d, *J* = 8.7), 7.31 (2H, d, *J* = 8.3), 8.35 (1H, s). *δ*_C_ (75 MHz, D_2_O): 19.2, 37.8, 39.1, 40.3, 53.7, 59.7, 63.9, 132.9, 133.3, 136.7, 137.6, 172.6, 173.0, 199.6. MS: C_16_H_22_ClN_3_O_4_S *m*/*z* (ES^+^) 388.1 [M + H^+^]. HRMS: Calculated for C_16_H_23_ClN_3_O_4_S 388.1098, found 388.1102.

#### 5.3.16 *(E)*-((*S*)-3-*tert*-butoxy-2-((*S*)-2-(*tert*-butoxycarbonyl)propanethioamido)-3-oxopropyl) 6-(4-hydroxy-6-methoxy-7-methyl-3-oxo-1,3-dihydroisobenzofuran-5-yl)-4-methylhex-4-enoate (**20**)

A mixture of **12** (94 mg, 0.27 mmol), mycophenolic acid (100 mg, 0.31 mmol) and *N*-methylmorpholine (137 mg, 1.24 mmol) in anhydrous MeCN (10 ml) was stirred with 3 Å molecular sieves under nitrogen at 20 °C for 2 h, then a solution of HATU (130 mg, 0.34 mmol) in MeCN (5 ml) was added and stirring was continued for another 2 days. The reaction was monitored by TLC (7:3 Petrol: EtOAC). The reaction mixture were filtered off and the filtrate, plus an MeCN washing, was evaporated in *vacuo* to get crude solid, which can further diluted with 30 ml of EtOAc, and washed with 2 M HCl (2 × 10 ml), saturated aqueous NaHCO_3_ (3 × 10 ml), Water (2 × 10 ml) and finally with brine (20 ml), dried (MgSO_4_) and evaporated in *vacuo* to afford white crude solid, which was further purified by chromatography, eluting with 9:1 (Petrol: EtOAC) to 7.5:1 (Petrol: EtOAC) to give desired product as a white solid (40 mg, 20%). M. p. 69–72 °C. R_*f*_ 0.50 [Petrol-EtOAc 1:1]. [α]_D_^20^ (CHCl_3_; c = 0.028): 35.71. υ_max_ (neat)/cm^−1^: 3326, 2976, 2930, 2115, 1991, 1868, 1738, 1732, 1716, 1699, 1622, 1564, 1506, 1454, 1411, 1393, 1367, 1329, 1245, 1179, 1130, 1049, 1038, 994, 969, 844, 792, 545. *δ*_H_ (300 MHz, CDCl_3_): 1.42 (21H, s), 1.73 (3H, s), 2.14 (3H, s), 2.22 (2H, t, *J* = 6.8), 2.25–2.35 (2H, m), 3.32 (2H, d, *J* = 6.8), 3.74 (3H, s), 4.35–4.53 (3H, m), 5.12–5.23 (4H, m), 7.62 (1H, s), 8.53 (1H, m). *δ*_C_ (75 MHz, CDCl_3_): 11.6, 16.1, 21.7, 22.5, 27.8, 28.2, 32.6, 34.3, 57.3, 61.0, 62.8, 70.1, 80.3, 83.5, 106.4, 116.7, 122.02, 122.8, 133.8, 144.1, 153.5, 163.6, 167.3, 172.8, 172.9, 205.6. MS: C_32_H_46_N_2_O_10_ S *m*/*z* (ES^+^) 651.3 [M + H^+^]. HRMS: Calculated for C_32_H_47_N_2_O_10_S 651.2951, found 651.2928.

#### 5.3.17 (*S*)-2-((*S*)-2-aminopropanethioamido)-3-((*E*)-6-(4-hydroxy-6-methoxy-7-methyl-3-oxo-1,3-dihydroisobenzofuran-5-yl)-4-methylhex-4-enoyloxy)propanoic acid (**11**)

Deprotection of 100 mg of **20** as for compound **8**. Yield of **11** as formate salt = 50 mg; 65%. M. p. 140–143 °C. [α]_D_^29^ (H_2_O; c = 0.057): −35.09. υ_max_ (neat)/cm^−1^: 3746, 3219, 2974, 2934, 2247, 2119, 1830, 1737, 1731, 1688, 1668, 161, 1606, 1558, 1539, 1532, 1516, 1452, 1409, 1380, 1325, 1303, 1251, 1187, 1135, 1107, 1075, 1033, 966, 938, 788, 642, 589, 542. *δ*_H_ (300 MHz, D_2_O): 1.22 (3H, d, *J* = 3.8), 1.43 (3H, s), 1.62–1.75 (3H, m), 1.91 (3H, s), 2.31–2.42 (2H, m), 2.52–2.63 (2H, m), 2.73–2.81 (1H, m), 3.02–3.11 (1H, m), 3.72 (3H, s), 4.21–4.43 (4H, m), 5.02–5.11 (3H, m), 8.01 (1H, s). *δ*_C_ (75 MHz, CD_3_OD): 11.4, 16.5, 20.5, 31.8, 33.2, 55.0, 62.9, 64.0, 70.8, 83.1, 120.0, 123.7, 124.2, 135.0, 162.5, 166.1, 170.8, 173.0, 201.2. MS: C_23_H_30_N_2_O_8_S *m*/*z* (ES^+^) 494.2 [M^+^]. HRMS: Calculated for C_23_H_30_N_2_O_8_S 494.1723, found 494.1676.

## Appendix A Supplementary data

SupplementaryInformation SupplementaryInformation
