# Supplementary material for: Thiodipeptides targeting the intestinal oligopeptide transporter as a general approach to improving oral drug delivery
Source: Eur J Med Chem. 2018 Aug 5;156:180–9. doi: 10.1016/j.ejmech.2018.06.064 (PMC6107785; doi:10.1016/j.ejmech.2018.06.064)
Supplement: SupplementaryInformation [file mmc1.pdf]

Reproduced NMR Spectra for Key Compounds

20 1H NMR

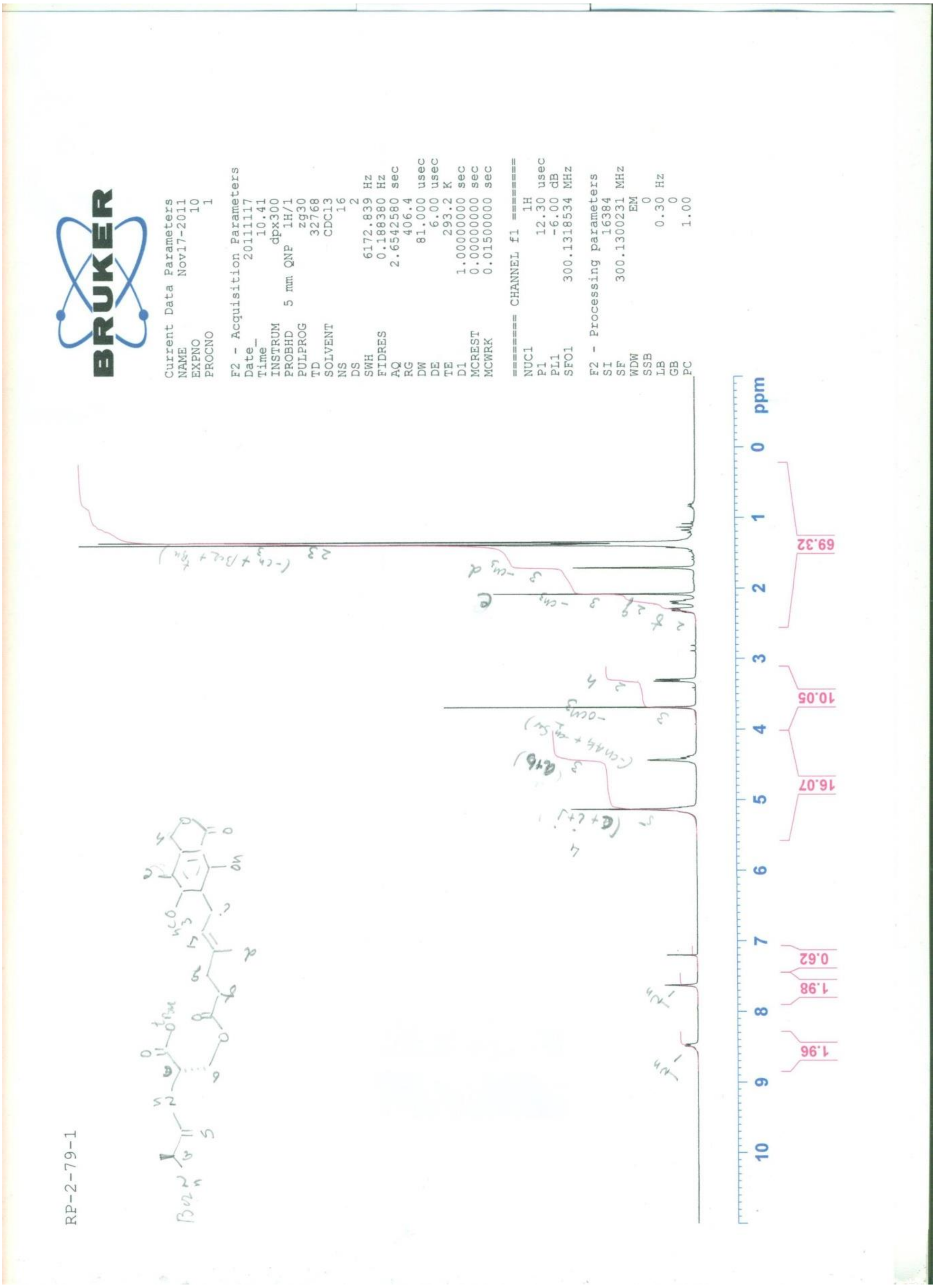

20 13C NMR

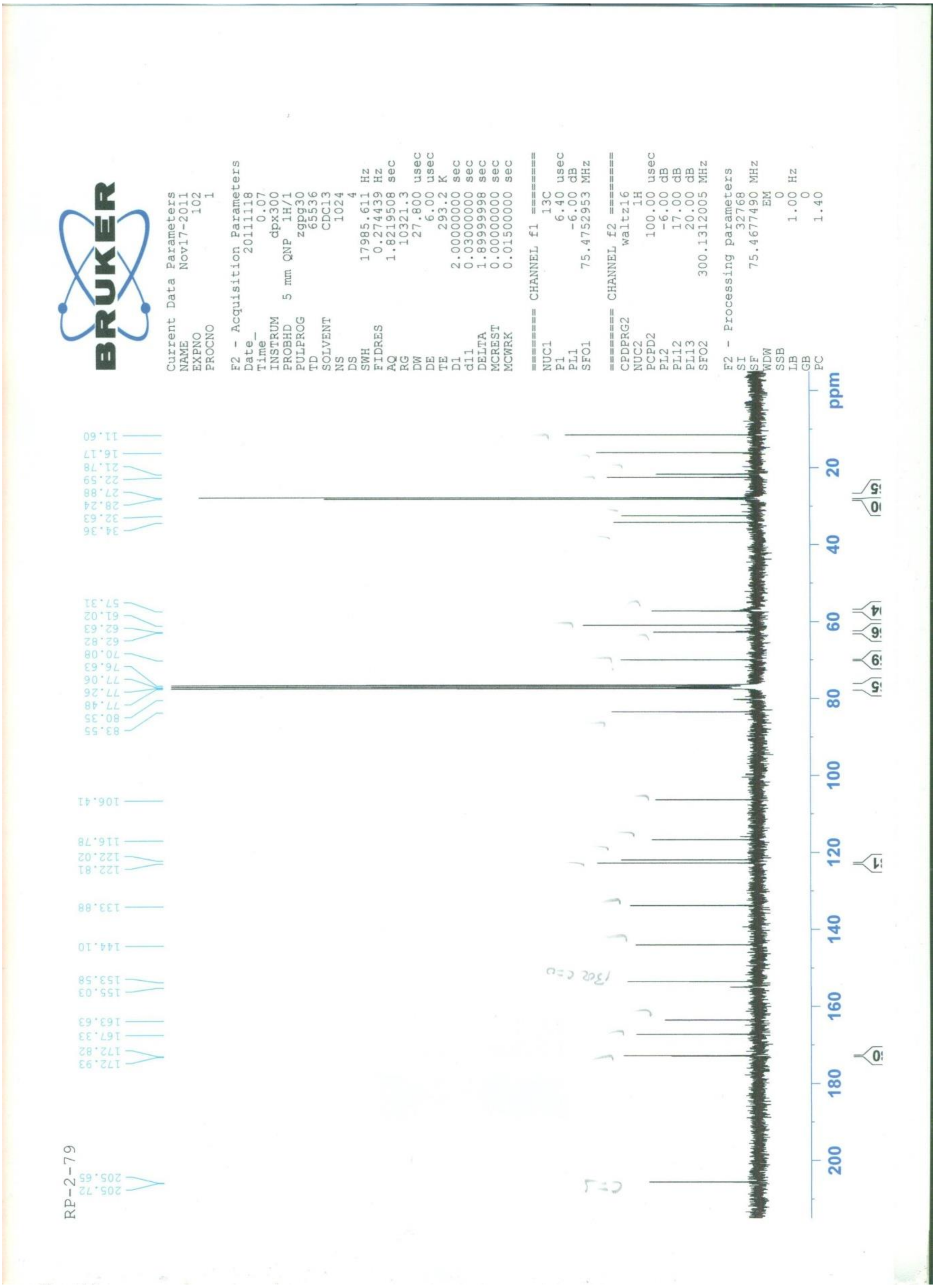
RP-2-79
11.60
16.17
21.78
22.59
27.88
28.24
32.63
34.36
57.31
61.02
62.63
62.82
70.08
76.63
77.06
77.26
77.48
80.35
83.55
106.41
116.78
122.02
122.81
133.88
144.10
153.58
155.03
163.63
167.33
172.82
172.93
205.65
205.72
BRUKER
Current Data Parameters
NAME Nov17-2011
EXPNO 102
PROCNO 1
F2 - Acquisition Parameters
Date_ 20111118
Time_ 0.07
INSTRUM dpx300
PROBHD 5 mm QNP 1H/1
PULPROG zgpg30
TD 65536
SOLVENT CDCl3
NS 1024
DS 4
SWH 17985.611 Hz
FIDRES 0.274439 Hz
AQ 1.8219508 sec
RG 10321.3
DW 27.800 usec
DE 6.00 usec
TE 293.2 K
D1 2.00000000 sec
d11 0.03000000 sec
DELTA 1.89999998 sec
MCREST 0.00000000 sec
MCWRK 0.01500000 sec
======== CHANNEL f1 ========
NUC1 13C
P1 6.40 usec
PL1 -6.00 dB
SFO1 75.4752953 MHz
======== CHANNEL f2 ========
CPDPRG2 waltz16
NUC2 1H
PCPD2 100.00 usec
PL2 -6.00 dB
PL12 17.00 dB
PL13 20.00 dB
SFO2 300.1312005 MHz
F2 - Processing parameters
SI 32768
SF 75.4677490 MHz
WDW EM
SSB 0
LB 1.00 Hz
GB 0
PC 1.40
ppm
20
40
60
80
100
120
140
160
180
200

11 1H NMR

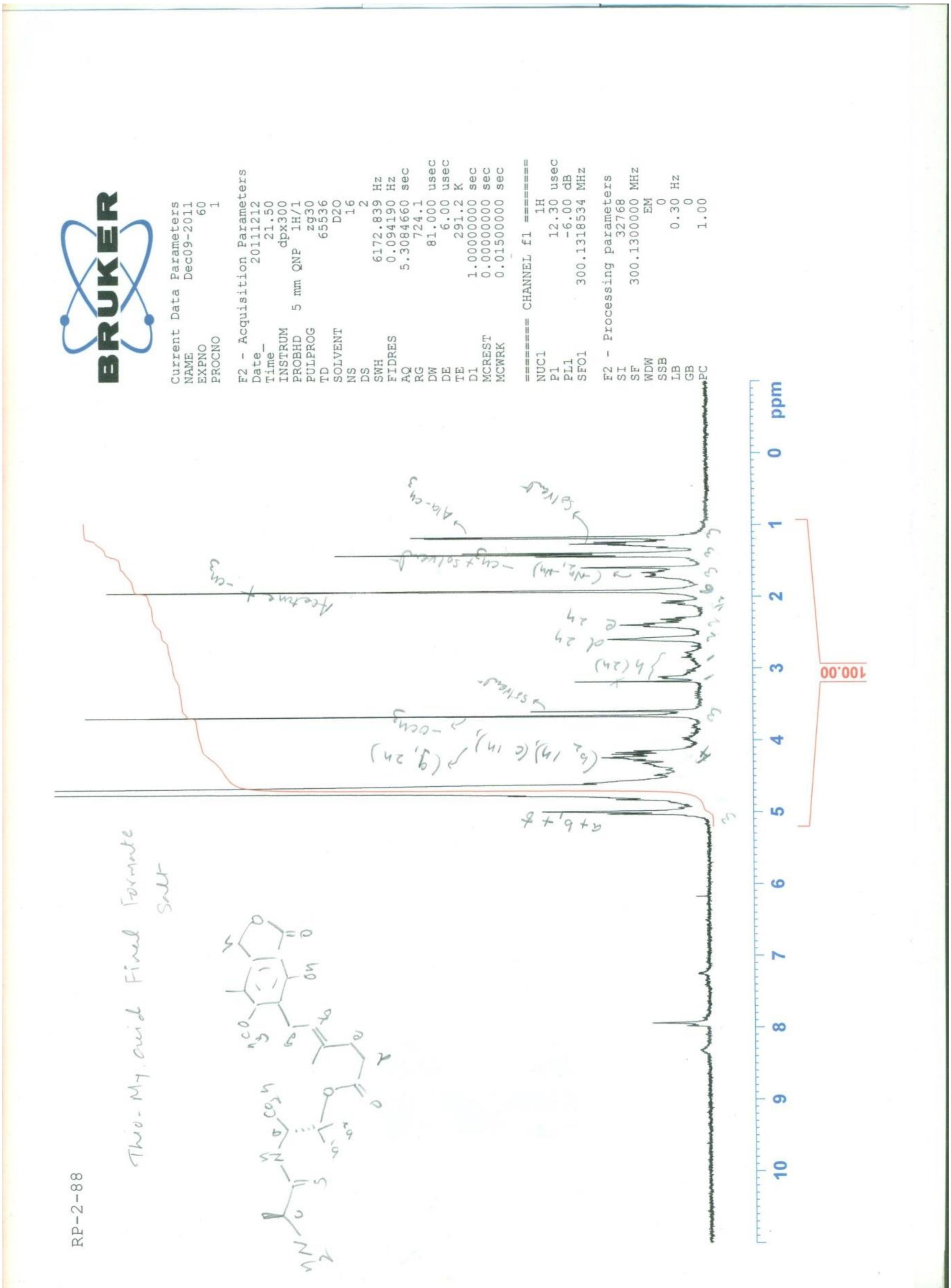

RP-2-88
Current Data Parameters
NAME Dec09-2011
EXPNO 60
PROCNO 1
F2 - Acquisition Parameters
Date_ 20111212
Time 21.50
INSTRUM dpx300
PROBHD 5 mm QNP 1H/1
PULPROG zg30
TD 65536
SOLVENT D2O
NS 16
DS 2
SWH 6172.839 Hz
FIDRES 0.094190 Hz
AQ 5.3084660 sec
RG 724.1
DW 81.000 usec
DE 6.00 usec
TE 291.2 K
D1 1.00000000 sec
MCREST 0.00000000 sec
MCWRK 0.01500000 sec
======== CHANNEL f1 ========
NUC1 1H
P1 12.30 usec
PL1 -6.00 dB
SFO1 300.1318534 MHz
F2 - Processing parameters
SI 32768
SF 300.1300000 MHz
WDW EM
SSB 0
LB 0.30 Hz
GB 0
PC 1.00
100.00
ppm

19 1H NMR

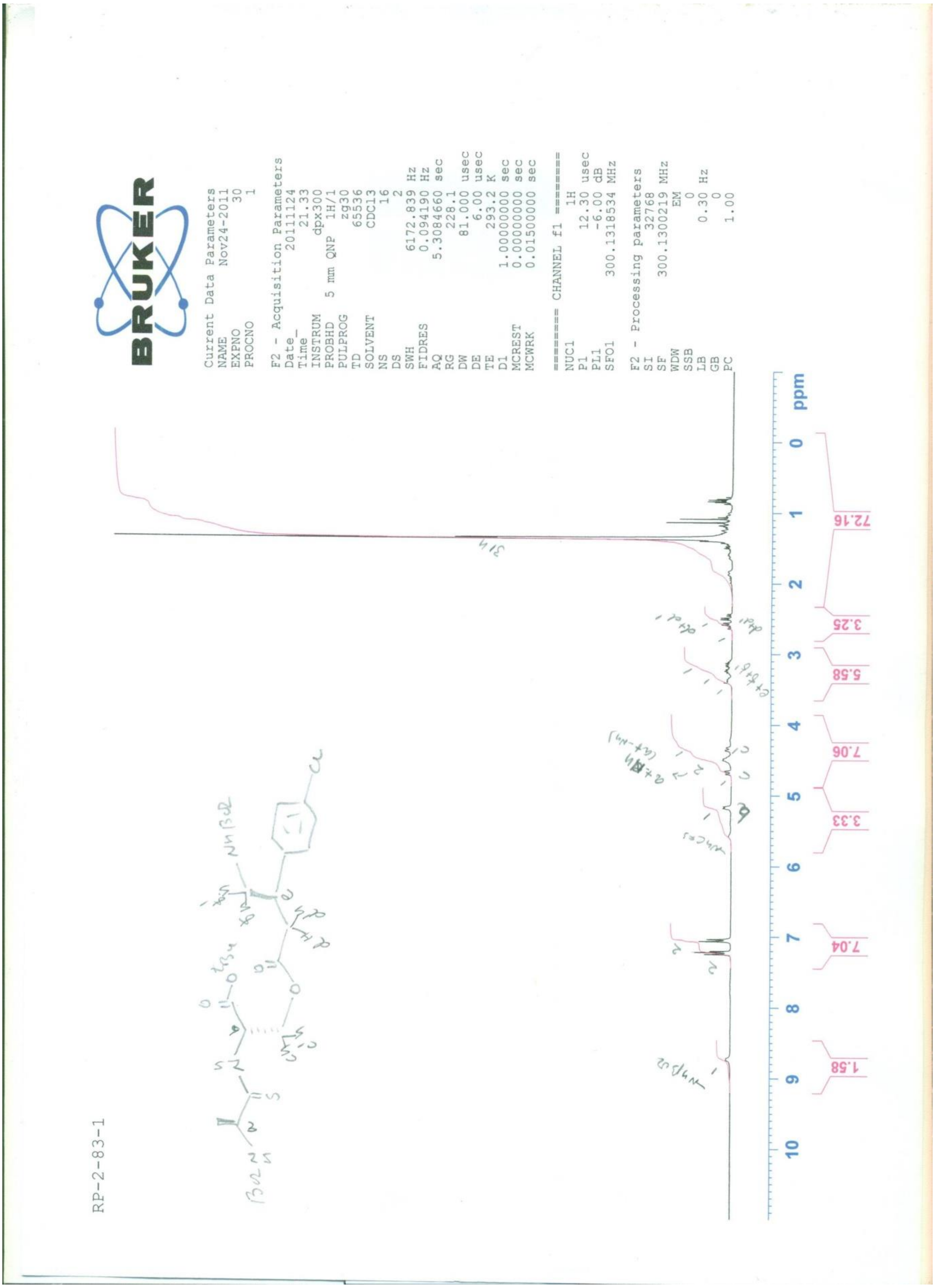
BRUKER
Current Data Parameters
NAME Nov24-2011
EXPNO 30
PROCNO 1
F2 - Acquisition Parameters
Date_ 20111124
Time 21.33
INSTRUM dpx300
PROBHD 5 mm QNP 1H/1
PULPROG zg30
TD 65536
SOLVENT CDCl3
NS 16
DS 2
SWH 6172.839 Hz
FIDRES 0.094190 Hz
AQ 5.3084660 sec
RG 228.1
DW 81.000 usec
DE 6.00 usec
TE 293.2 K
D1 1.00000000 sec
MCREST 0.00000000 sec
MCWRK 0.01500000 sec
======== CHANNEL f1 ========
NUC1 1H
P1 12.30 usec
PL1 -6.00 dB
SFO1 300.1318534 MHz
F2 - Processing parameters
SI 32768
SF 300.1300219 MHz
WDW EM
SSB 0
LB 0.30 Hz
GB 0
PC 1.00
RP-2-83-1
72.16
3.25
5.58
7.06
3.33
7.04
1.58
10 9 8 7 6 5 4 3 2 1 0 ppm

19 13C NMR

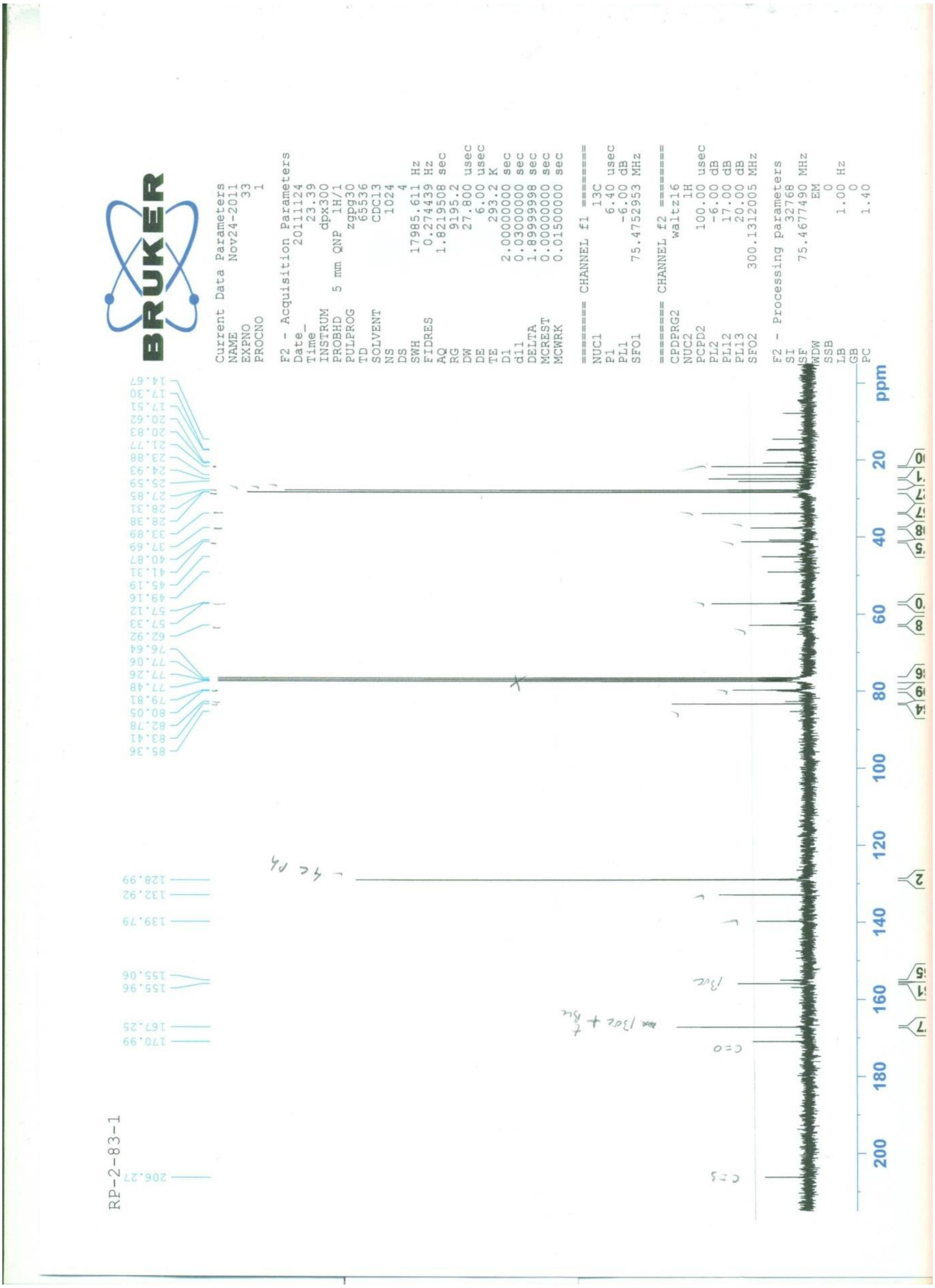
RP-2-83-1
206.27
170.99
167.25
155.96
155.06
139.79
132.92
128.99
85.36
83.41
82.78
80.05
79.81
77.48
77.26
77.06
76.64
62.92
57.33
57.12
49.16
45.19
41.31
40.87
37.69
33.89
28.38
28.31
27.85
25.59
24.93
23.88
21.77
20.83
20.62
17.51
17.30
14.67
ppm
Current Data Parameters
NAME Nov24-2011
EXPNO 33
PROCNO 1
F2 - Acquisition Parameters
Date_ 20111124
Time 23.39
INSTRUM dpx300
PROBHD 5 mm QNP 1H/1
PULPROG zgpg30
TD 65536
SOLVENT CDCl3
NS 1024
DS 4
SWH 17985.611 Hz
FIDRES 0.274439 Hz
AQ 1.8219508 sec
RG 9195.2
DW 27.800 usec
DE 6.00 usec
TE 293.2 K
D1 2.00000000 sec
d11 0.03000000 sec
DELTA 1.89999998 sec
MCREST 0.00000000 sec
MCWRK 0.01500000 sec
======== CHANNEL f1 ========
NUC1 13C
P1 6.40 usec
PL1 -6.00 dB
SFO1 75.4752953 MHz
======== CHANNEL f2 ========
CPDPRG2 waltz16
NUC2 1H
PCPD2 100.00 usec
PL2 -6.00 dB
PL12 17.00 dB
PL13 20.00 dB
SFO2 300.1312005 MHz
F2 - Processing parameters
SI 32768
SF 75.4677490 MHz
WDW EM
SSB 0
LB 1.00 Hz
GB 0
PC 1.40

10 1H NMR

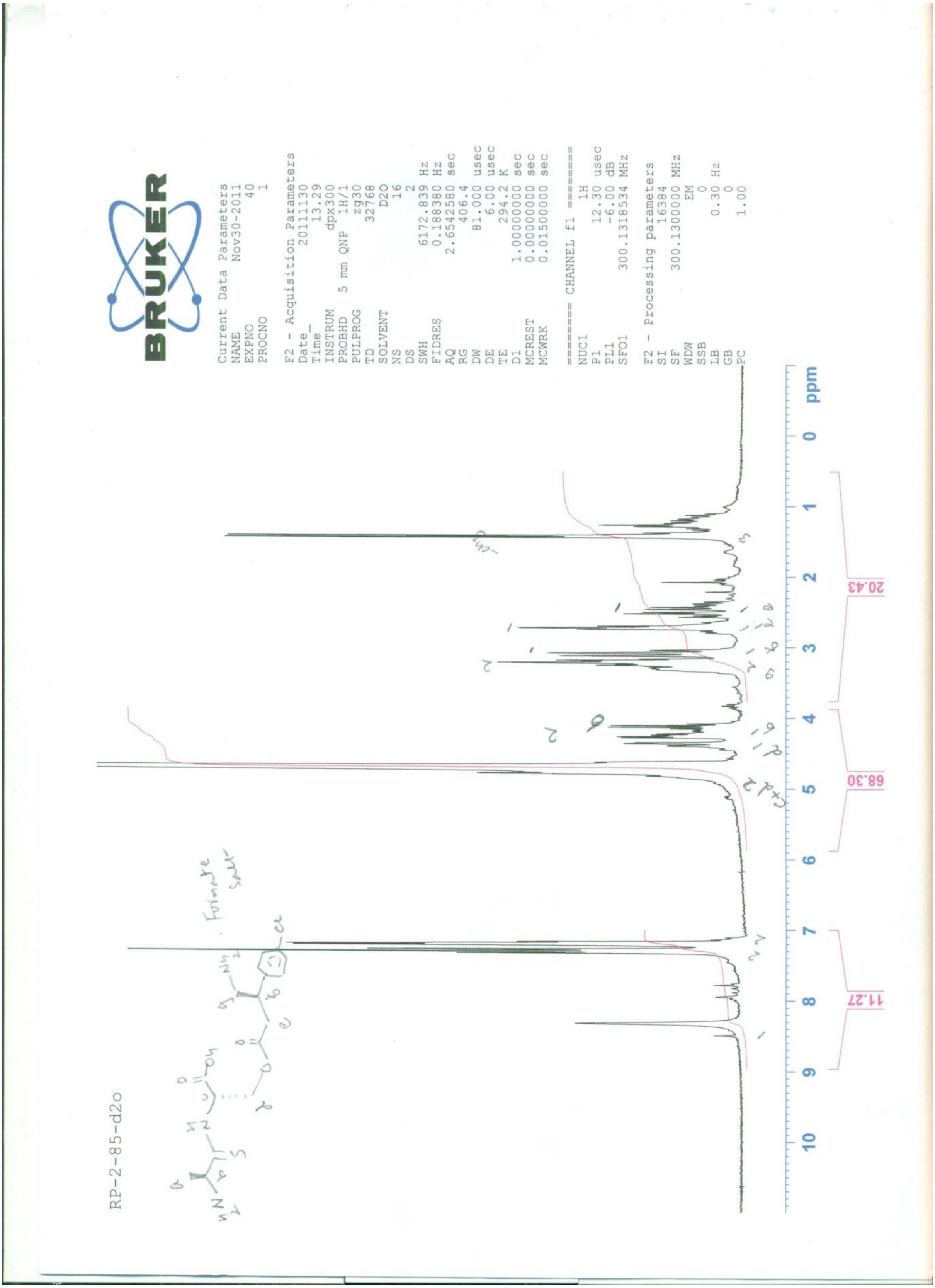
RP-2-85-d2o
BRUKER
Current Data Parameters
NAME Nov30-2011
EXPNO 40
PROCNO 1
F2 - Acquisition Parameters
Date_ 20111130
Time_ 13.29
INSTRUM dpx300
PROBHD 5 mm QNP 1H/1
PULPROG zg30
TD 32768
SOLVENT D2O
NS 16
DS 2
SWH 6172.839 Hz
FIDRES 0.188380 Hz
AQ 2.6542580 sec
RG 406.4
DW 81.000 usec
DE 6.00 usec
TE 294.2 K
D1 1.00000000 sec
MCREST 0.00000000 sec
MCWRK 0.01500000 sec
======== CHANNEL f1 ========
NUC1 1H
P1 12.30 usec
PL1 -6.00 dB
SFO1 300.1318534 MHz
F2 - Processing parameters
SI 16384
SF 300.1300000 MHz
WDW EM
SSB 0
LB 0.30 Hz
GB 0
PC 1.00
20.43
68.30
11.27
ppm

10 13C NMR

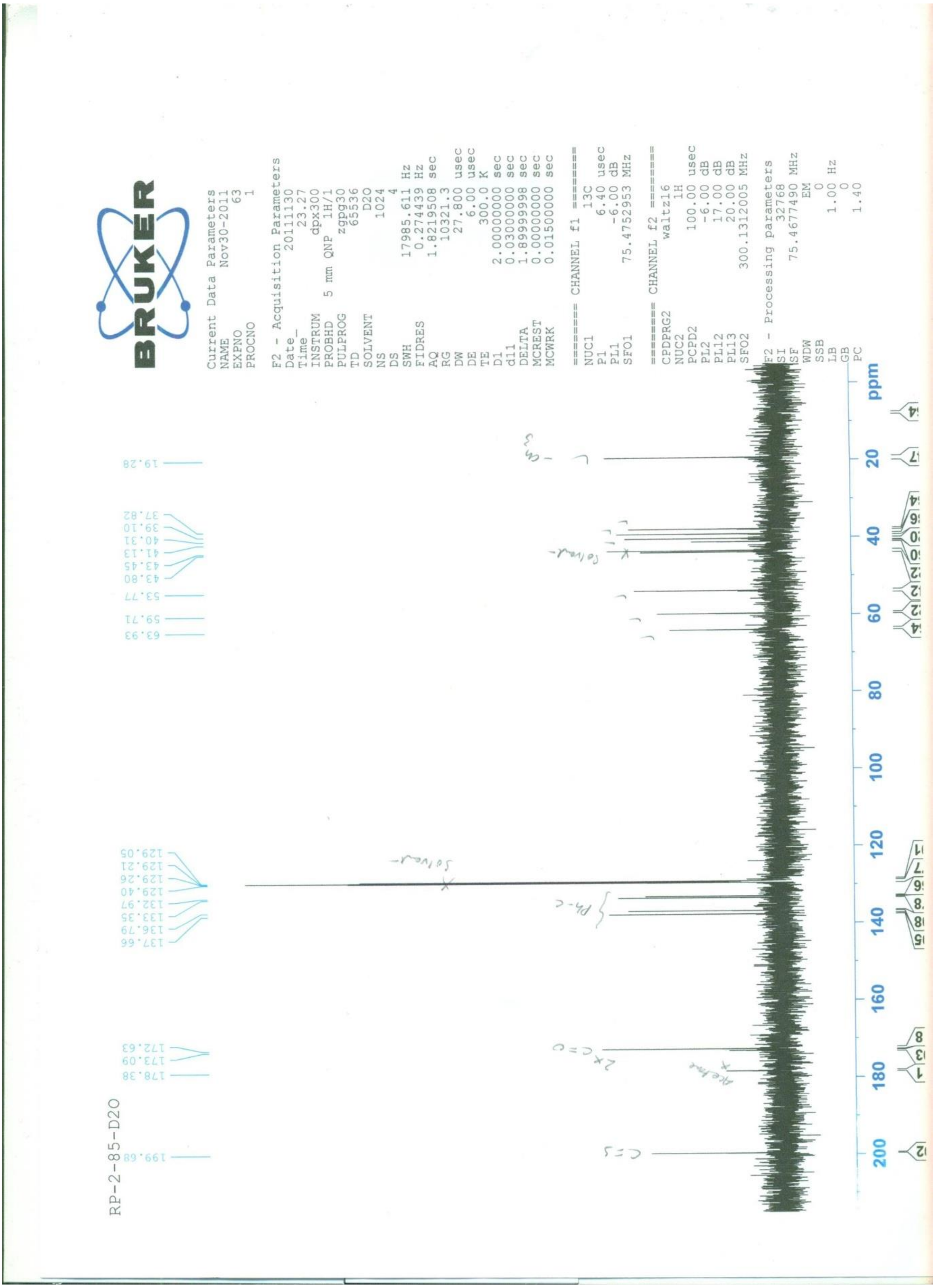

BRUKER
Current Data Parameters
NAME Nov30-2011
EXPNO 63
PROCNO 1
F2 - Acquisition Parameters
Date_ 20111130
Time 23.27
INSTRUM dpx300
PROBHD 5 mm QNP 1H/1
PULPROG zgpg30
TD 65536
SOLVENT D2O
NS 1024
DS 4
SWH 17985.611 Hz
FIDRES 0.274439 Hz
AQ 1.8219508 sec
RG 10321.3
DW 27.800 usec
DE 6.00 usec
TE 300.0 K
D1 2.00000000 sec
d11 0.03000000 sec
DELTA 1.89999998 sec
MCREST 0.00000000 sec
MCWRK 0.01500000 sec
======== CHANNEL f1 ========
NUC1 13C
P1 6.40 usec
PL1 -6.00 dB
SFO1 75.4752953 MHz
======== CHANNEL f2 ========
CPDPRG2 waltz16
NUC2 1H
PCPD2 100.00 usec
PL2 -6.00 dB
PL12 17.00 dB
PL13 20.00 dB
SFO2 300.1312005 MHz
F2 - Processing parameters
SI 32768
SF 75.4677490 MHz
WDW EM
SSB 0
LB 1.00 Hz
GB 0
PC 1.40
RP-2-85-D2O
19.28
37.82
39.10
40.31
41.13
43.45
43.80
53.77
59.71
63.93
129.05
129.21
129.26
129.40
132.47
132.95
133.35
136.79
137.66
172.63
173.09
178.38
199.68
ppm
20
40
60
80
100
120
140
160
180
200

17 1H NMR

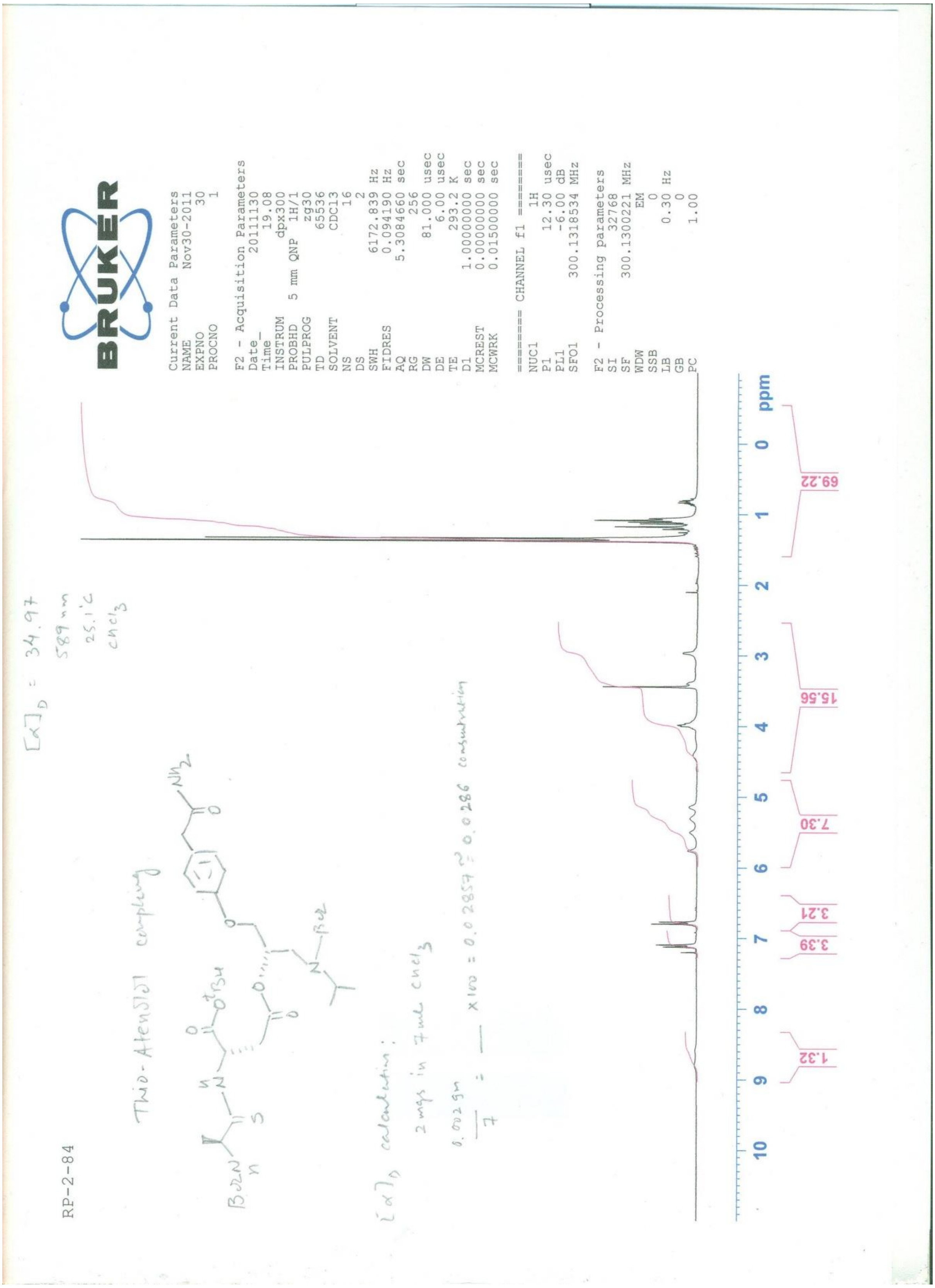

RP-2-84
BRUKER
Current Data Parameters
NAME Nov30-2011
EXPNO 30
PROCNO 1
F2 - Acquisition Parameters
Date_ 20111130
Time 19.08
INSTRUM dpx300
PROBHD 5 mm QNP 1H/1
PULPROG zg30
TD 65536
SOLVENT CDCl3
NS 16
DS 2
SWH 6172.839 Hz
FIDRES 0.094190 Hz
AQ 5.3084660 sec
RG 256
DW 81.000 usec
DE 6.00 usec
TE 293.2 K
D1 1.00000000 sec
MCREST 0.00000000 sec
MCWRK 0.01500000 sec
======== CHANNEL f1 ========
NUC1 1H
P1 12.30 usec
PL1 -6.00 dB
SFO1 300.1318534 MHz
F2 - Processing parameters
SI 32768
SF 300.1300221 MHz
WDW EM
SSB 0
LB 0.30 Hz
GB 0
PC 1.00
ppm
69.22
15.56
7.30
3.21
3.39
1.32

17 13C NMR

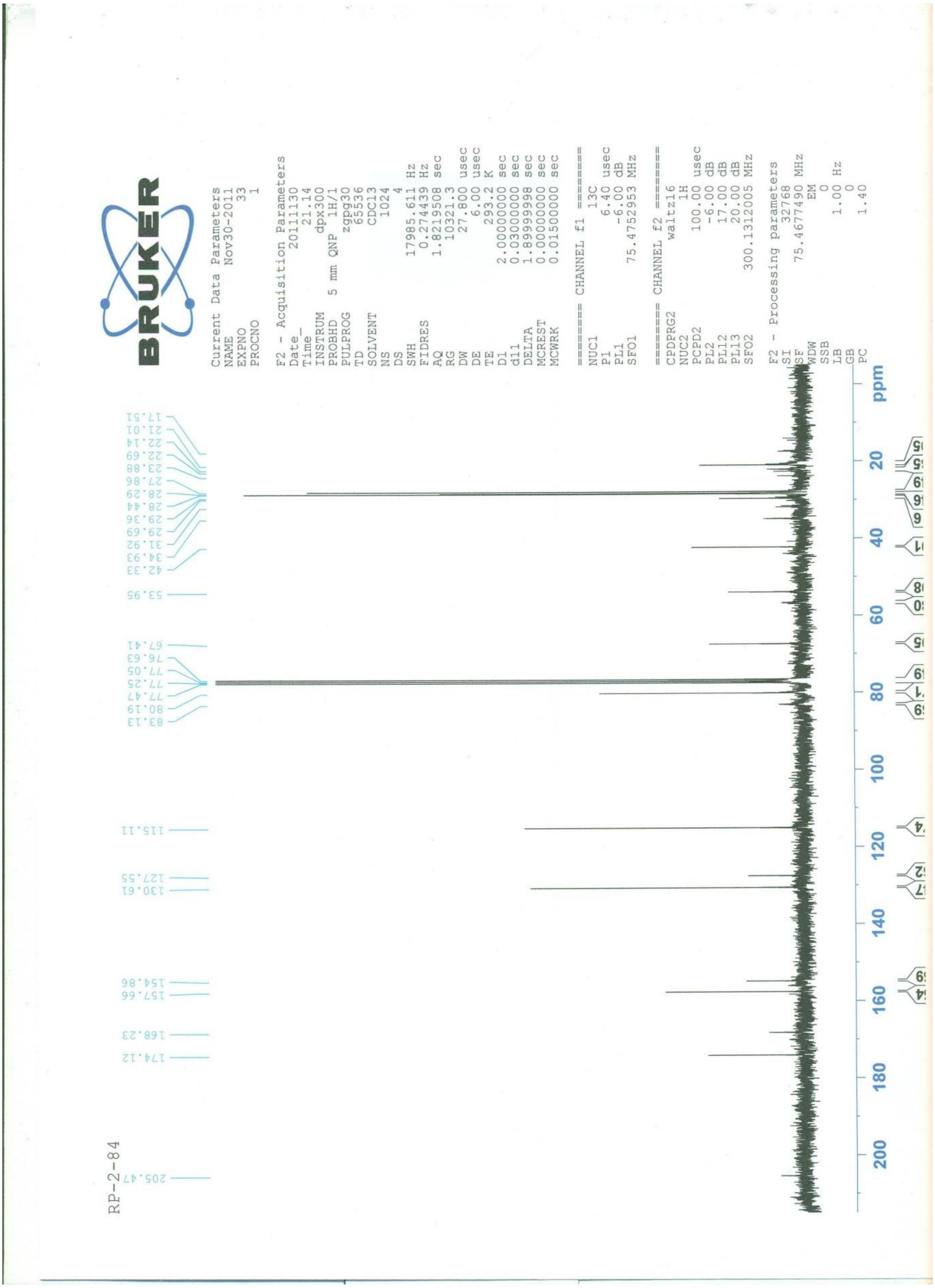
RP-2-84
17.51
21.01
22.14
22.69
23.88
27.86
28.29
28.44
29.36
29.69
31.92
34.93
42.33
53.95
67.41
76.63
77.05
77.25
77.47
80.19
83.13
115.11
127.55
130.61
154.86
157.66
168.23
174.12
205.47
BRUKER
Current Data Parameters
NAME Nov30-2011
EXPNO 33
PROCNO 1
F2 - Acquisition Parameters
Date_ 20111130
Time 21.14
INSTRUM dpx300
PROBHD 5 mm QNP 1H/1
PULPROG zgpg30
TD 65536
SOLVENT CDCl3
NS 1024
DS 4
SWH 17985.611 Hz
FIDRES 0.274439 Hz
AQ 1.8219508 sec
RG 10321.3
DW 27.800 usec
DE 6.00 usec
TE 293.2 K
D1 2.00000000 sec
d11 0.03000000 sec
DELTA 1.89999998 sec
MCREST 0.00000000 sec
MCWRK 0.01500000 sec
======== CHANNEL f1 ========
NUC1 13C
P1 6.40 usec
PL1 -6.00 dB
SFO1 75.4752953 MHz
======== CHANNEL f2 ========
CPDPRG2 waltz16
NUC2 1H
PCPD2 100.00 usec
PL2 -6.00 dB
PL12 17.00 dB
PL13 20.00 dB
SFO2 300.1312005 MHz
F2 - Processing parameters
SI 32768
SF 75.4677490 MHz
WDW EM
SSB 0
LB 1.00 Hz
GB 0
PC 1.40
200
180
160
140
120
100
80
60
40
20
ppm

8 1H NMR

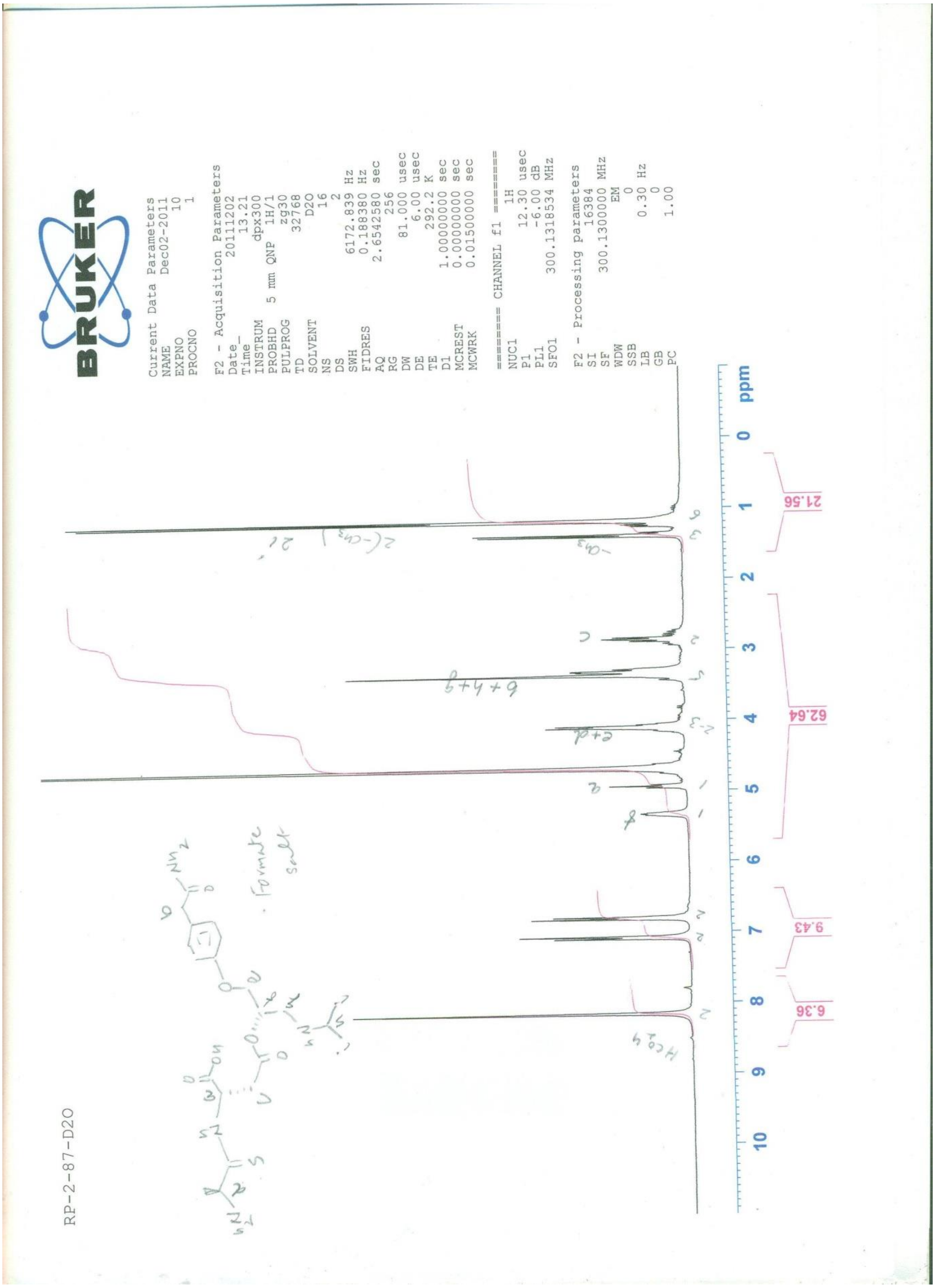
BRUKER
Current Data Parameters
NAME Dec02-2011
EXPNO 10
PROCNO 1
F2 - Acquisition Parameters
Date_ 20111202
Time 13.21
INSTRUM dpx300
PROBHD 5 mm QNP 1H/1
PULPROG zg30
TD 32768
SOLVENT D2O
NS 16
DS 2
SWH 6172.839 Hz
FIDRES 0.188380 Hz
AQ 2.6542580 sec
RG 256
DW 81.000 usec
DE 6.00 usec
TE 292.2 K
D1 1.00000000 sec
MCREST 0.00000000 sec
MCWRK 0.01500000 sec
======== CHANNEL f1 ========
NUC1 1H
P1 12.30 usec
PL1 -6.00 dB
SFO1 300.1318534 MHz
F2 - Processing parameters
SI 16384
SF 300.1300000 MHz
WDW EM
SSB 0
LB 0.30 Hz
GB 0
PC 1.00
RP-2-87-D2O
ppm
10 9 8 7 6 5 4 3 2 1 0
6.36
9.43
62.64
21.56

8 13C NMR

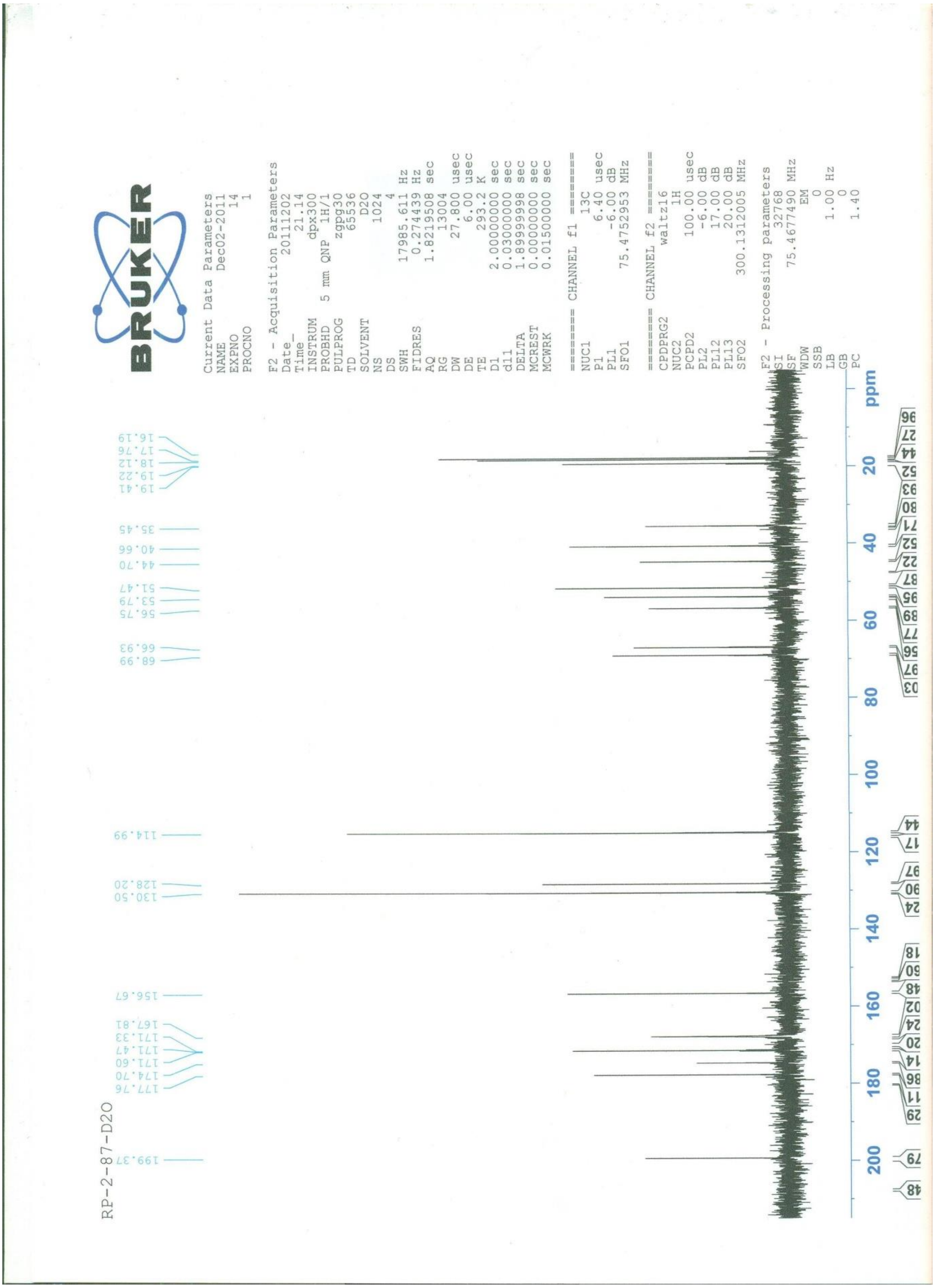
RP-2-87-D2O
16.19
17.76
18.12
19.22
19.41
35.45
40.66
44.70
51.47
53.79
56.75
66.93
68.99
114.99
128.20
130.50
156.67
167.81
171.33
171.47
171.60
174.70
177.76
199.37
BRUKER
Current Data Parameters
NAME Dec02-2011
EXPNO 14
PROCNO 1
F2 - Acquisition Parameters
Date_ 20111202
Time 21.14
INSTRUM dpx300
PROBHD 5 mm QNP 1H/1
PULPROG zgpg30
TD 65536
SOLVENT D2O
NS 1024
DS 4
SWH 17985.611 Hz
FIDRES 0.274439 Hz
AQ 1.8219508 sec
RG 13004
DW 27.800 usec
DE 6.00 usec
TE 293.2 K
D1 2.00000000 sec
d11 0.03000000 sec
DELTA 1.89999998 sec
MCREST 0.00000000 sec
MCWRK 0.01500000 sec
======== CHANNEL f1 ========
NUC1 13C
P1 6.40 usec
PL1 -6.00 dB
SFO1 75.4752953 MHz
======== CHANNEL f2 ========
CPDPRG2 waltz16
NUC2 1H
PCPD2 100.00 usec
PL2 -6.00 dB
PL12 17.00 dB
PL13 20.00 dB
SFO2 300.1312005 MHz
F2 - Processing parameters
SI 32768
SF 75.4677490 MHz
WDW EM
SSB 0
LB 1.00 Hz
GB 0
PC 1.40
ppm
20
40
60
80
100
120
140
160
180
200

18 1H NMR

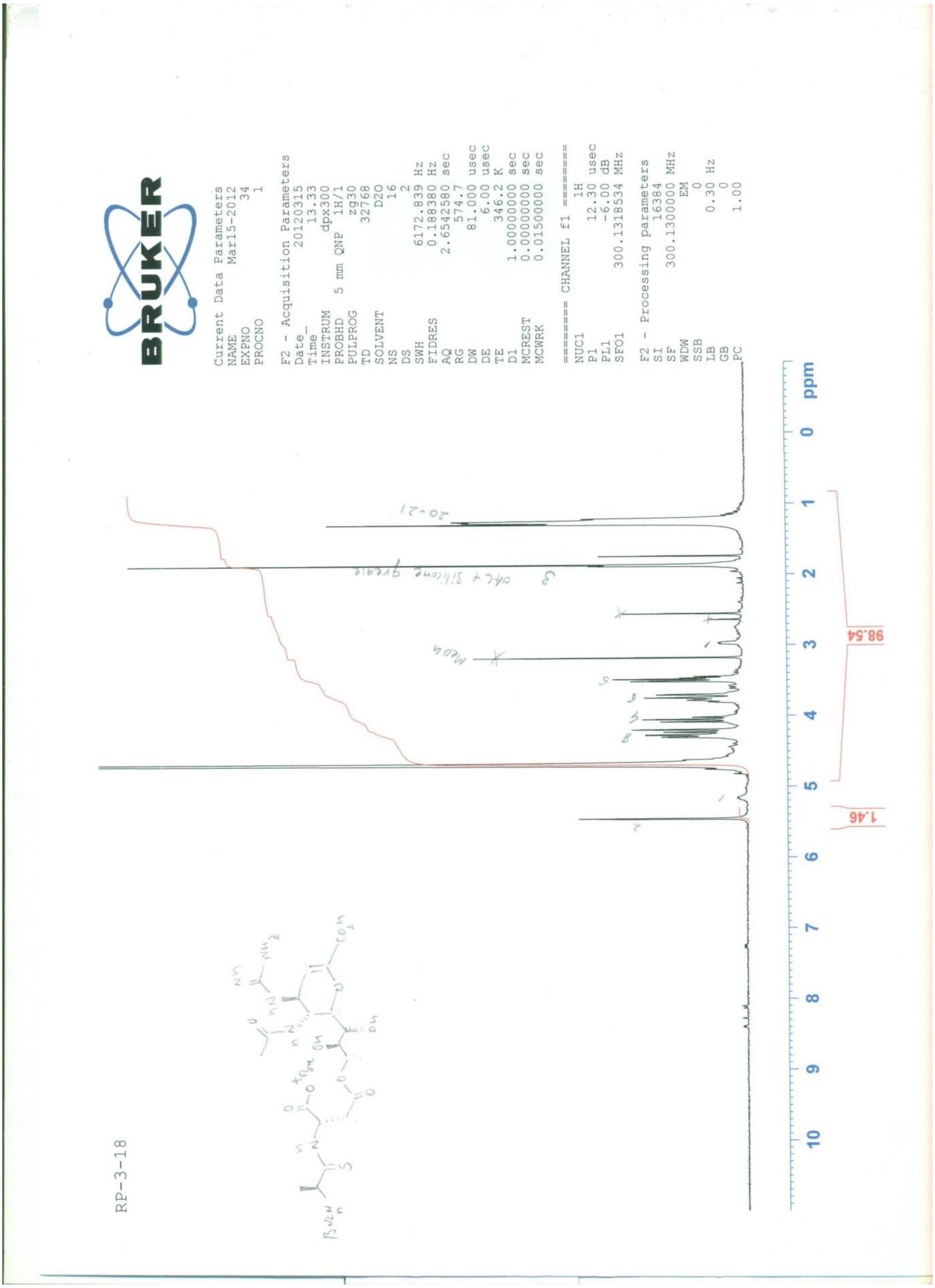
RP-3-18
BRUKER
Current Data Parameters
NAME Mar15-2012
EXPNO 34
PROCNO 1
F2 - Acquisition Parameters
Date_ 20120315
Time_ 13.33
INSTRUM dpx300
PROBHD 5 mm QNP 1H/1
PULPROG zg30
TD 32768
SOLVENT D2O
NS 16
DS 2
SWH 6172.839 Hz
FIDRES 0.188380 Hz
AQ 2.6542580 sec
RG 574.7
DW 81.000 usec
DE 6.00 usec
TE 346.2 K
D1 1.00000000 sec
MCREST 0.00000000 sec
MCWRK 0.01500000 sec
======== CHANNEL f1 ========
NUC1 1H
P1 12.30 usec
PL1 -6.00 dB
SFO1 300.1318534 MHz
F2 - Processing parameters
SI 16384
SF 300.1300000 MHz
WDW EM
SSB 0
LB 0.30 Hz
GB 0
PC 1.00
98.54
1.46
ppm
10 9 8 7 6 5 4 3 2 1 0

18 13C NMR

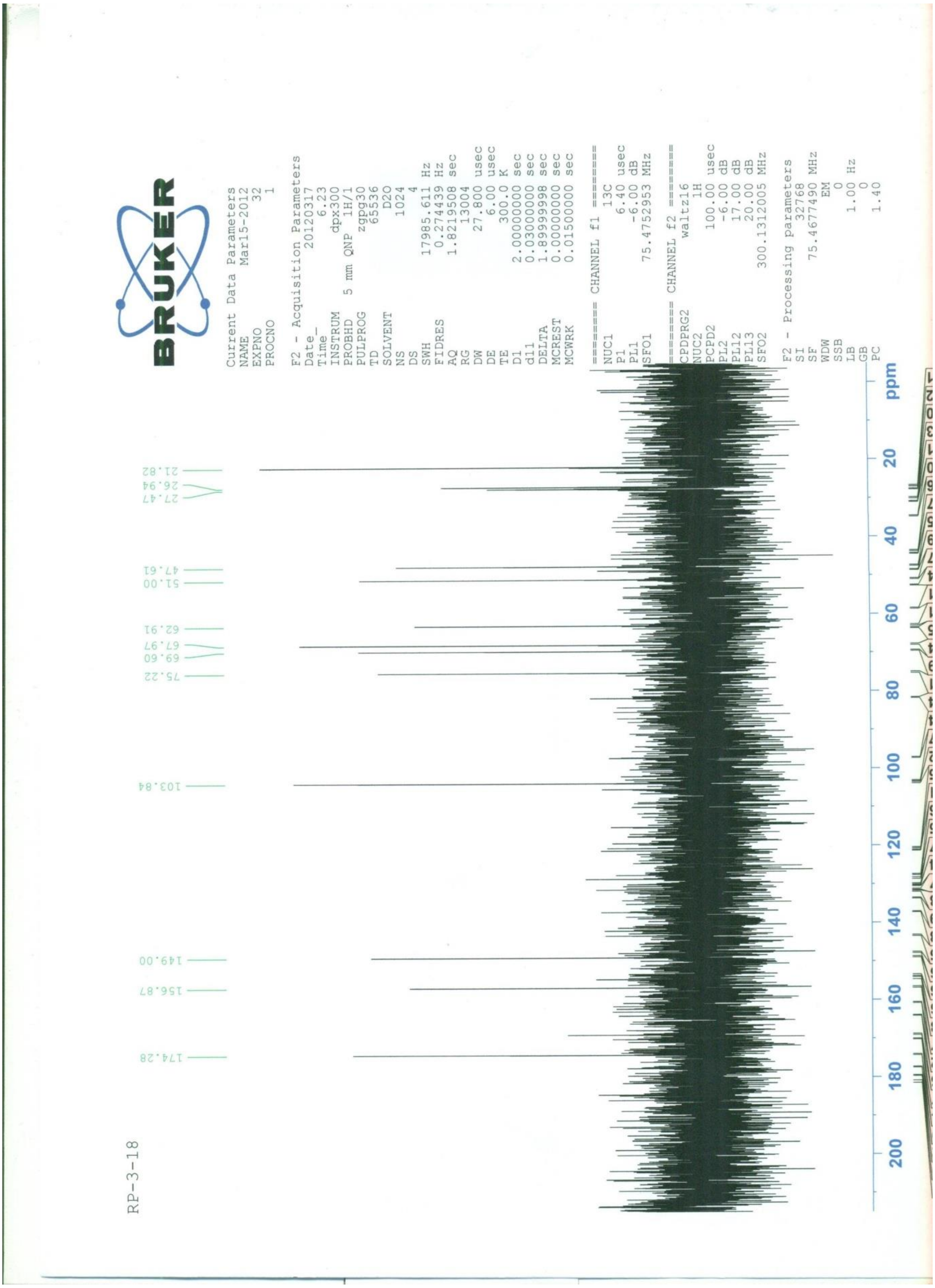
BRUKER
Current Data Parameters
NAME Mar15-2012
EXPNO 32
PROCNO 1
F2 - Acquisition Parameters
Date_ 20120317
Time 6.23
INSTRUM dpx300
PROBHD 5 mm QNP 1H/1
PULPROG zgpg30
TD 65536
SOLVENT D2O
NS 1024
DS 4
SWH 17985.611 Hz
FIDRES 0.274439 Hz
AQ 1.8219508 sec
RG 13004
DW 27.800 usec
DE 6.00 usec
TE 300.0 K
D1 2.00000000 sec
d11 0.03000000 sec
DELTA 1.89999998 sec
MCREST 0.00000000 sec
MCWRK 0.01500000 sec
======== CHANNEL f1 ========
NUC1 13C
P1 6.40 usec
PL1 -6.00 dB
SFO1 75.4752953 MHz
======== CHANNEL f2 ========
CPDPRG2 waltz16
NUC2 1H
PCPD2 100.00 usec
PL2 -6.00 dB
PL12 17.00 dB
PL13 20.00 dB
SFO2 300.1312005 MHz
F2 - Processing parameters
SI 32768
SF 75.4677490 MHz
WDW EM
SSB 0
LB 1.00 Hz
GB 0
PC 1.40
21.82
26.94
27.47
47.61
51.00
62.91
67.97
69.60
75.22
103.84
149.00
156.87
174.28
RP-3-18
ppm
20
40
60
80
100
120
140
160
180
200

9 1H NMR

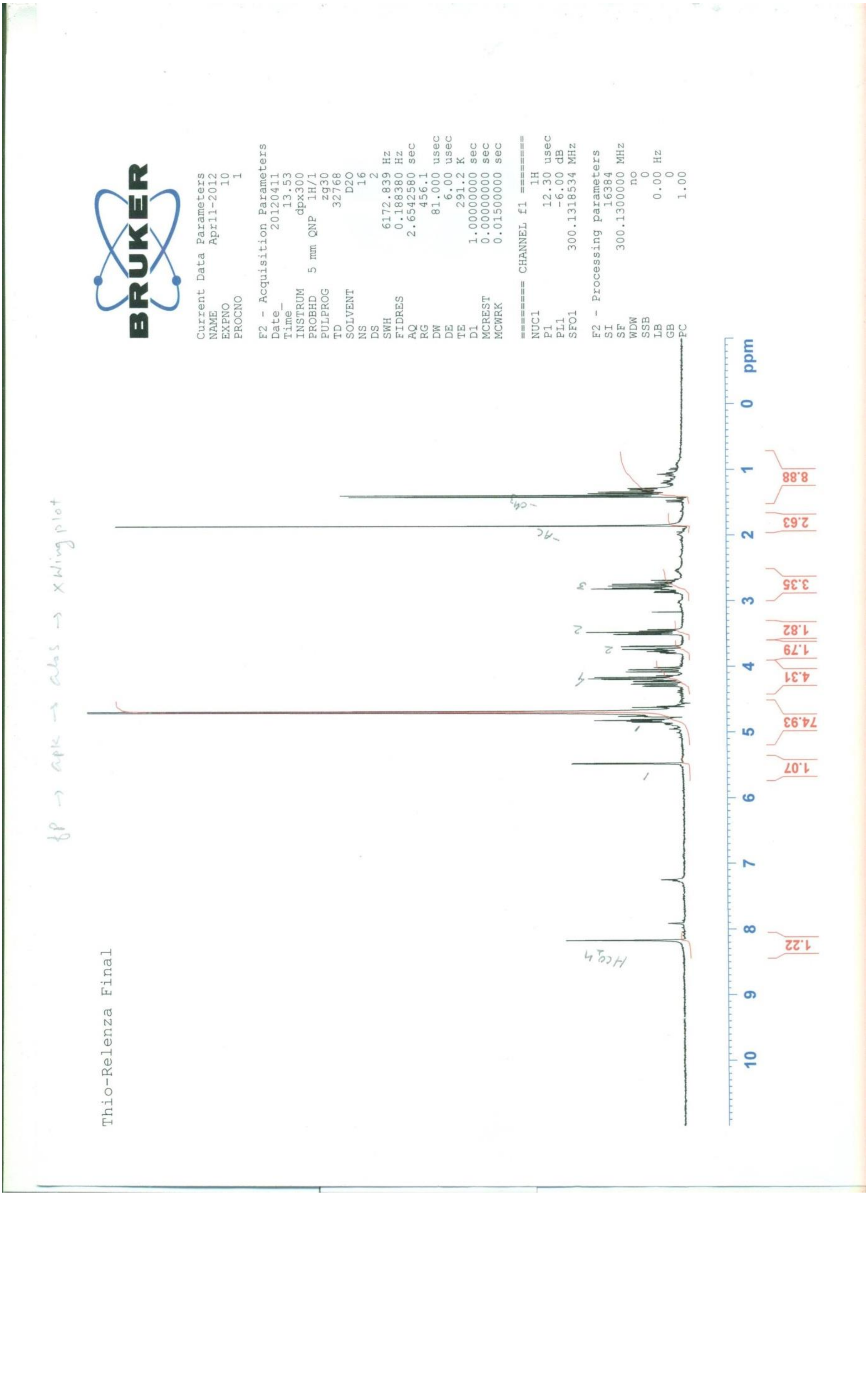

Thio-Relenza Final
BRUKER
Current Data Parameters
NAME Apr11-2012
EXPNO 10
PROCNO 1
F2 - Acquisition Parameters
Date_ 20120411
Time 13.53
INSTRUM dpx300
PROBHD 5 mm QNP 1H/1
PULPROG zg30
TD 32768
SOLVENT D2O
NS 16
DS 2
SWH 6172.839 Hz
FIDRES 0.188380 Hz
AQ 2.6542580 sec
RG 456.1
DW 81.000 usec
DE 6.00 usec
TE 291.2 K
D1 1.00000000 sec
MCREST 0.00000000 sec
MCWRK 0.01500000 sec
======== CHANNEL f1 ========
NUC1 1H
P1 12.30 usec
PL1 -6.00 dB
SFO1 300.1318534 MHz
F2 - Processing parameters
SI 16384
SF 300.1300000 MHz
WDW no
SSB 0
LB 0.00 Hz
GB 0
PC 1.00
ppm
1.22
1.07
74.93
4.31
1.79
1.82
3.35
2.63
8.88

9 13C NMR

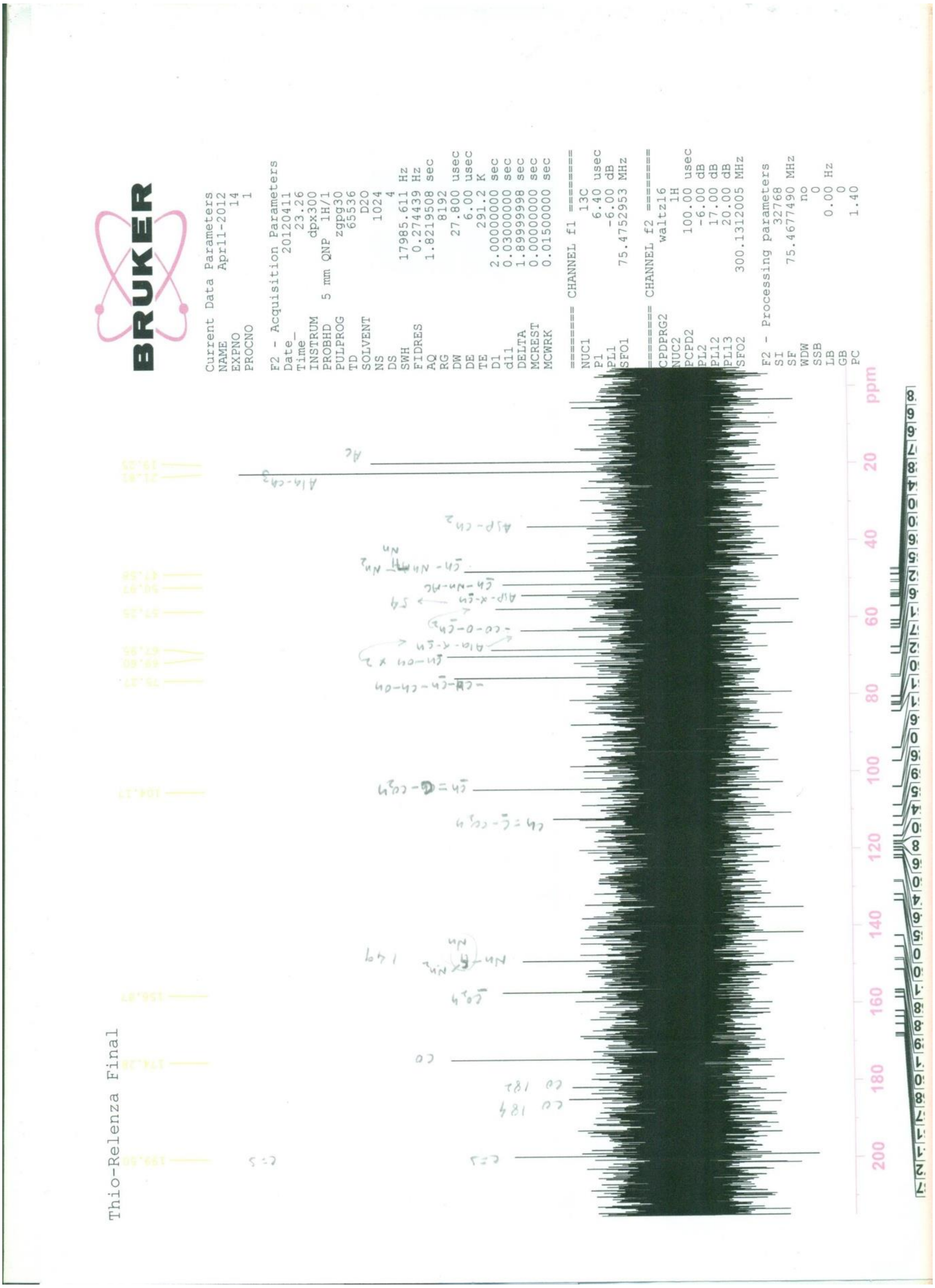

Thio-Relenza Final
BRUKER
Current Data Parameters
NAME Apr11-2012
EXPNO 14
PROCNO 1
F2 - Acquisition Parameters
Date_ 20120411
Time_ 23.26
INSTRUM dpx300
PROBHD 5 mm QNP 1H/1
PULPROG zgpg30
TD 65536
SOLVENT D2O
NS 1024
DS 4
SWH 17985.611 Hz
FIDRES 0.274439 Hz
AQ 1.8219508 sec
RG 8192
DW 27.800 usec
DE 6.00 usec
TE 291.2 K
D1 2.00000000 sec
d11 0.03000000 sec
DELTA 1.89999998 sec
MCREST 0.00000000 sec
MCWRK 0.01500000 sec
======== CHANNEL f1 ========
NUC1 13C
P1 6.40 usec
PL1 -6.00 dB
SFO1 75.4752953 MHz
======== CHANNEL f2 ========
CPDPRG2 waltz16
NUC2 1H
PCPD2 100.00 usec
PL2 -6.00 dB
PL12 17.00 dB
PL13 20.00 dB
SFO2 300.1312005 MHz
F2 - Processing parameters
SI 32768
SF 75.4677490 MHz
WDW no
SSB 0
LB 0.00 Hz
GB 0
PC 1.40
ppm

4 1H NMR

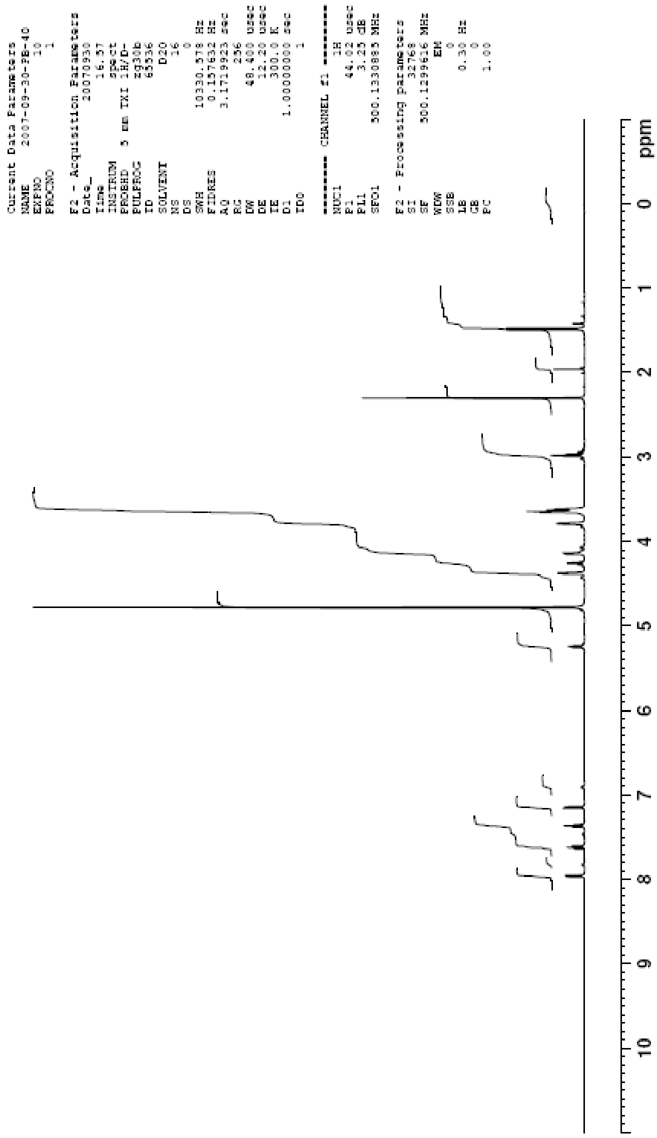

5 1H NMR

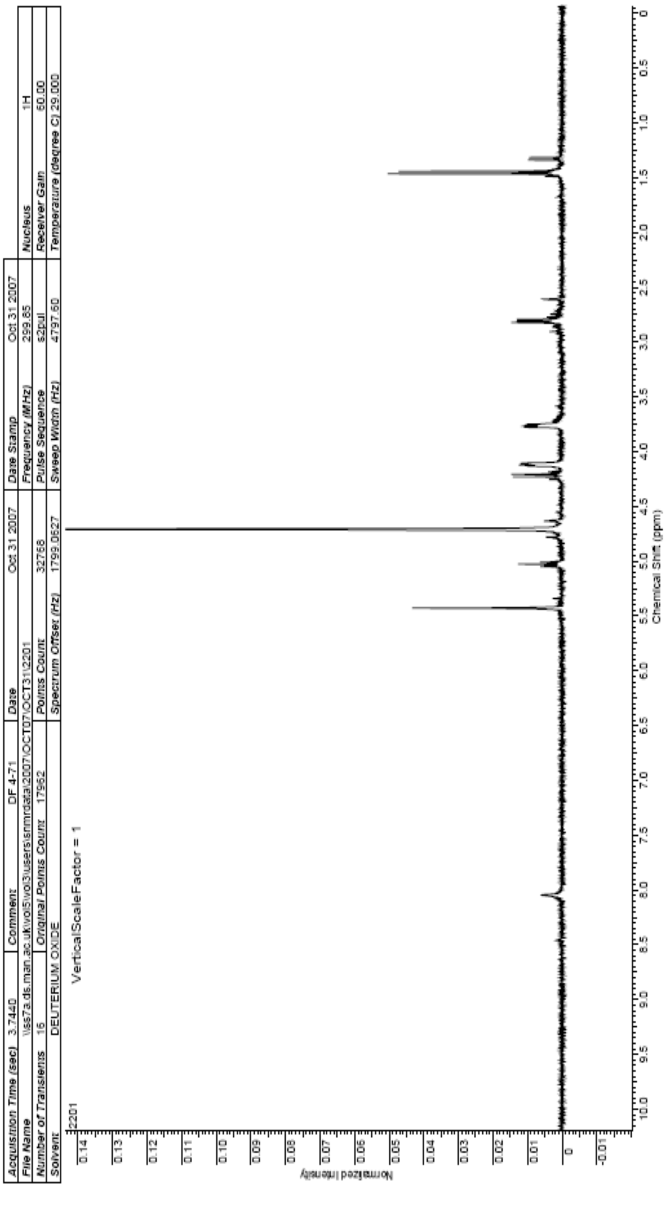

Acquisition Time (sec) 3.7440
Comment DF 4-71
Date Oct 31 2007
Date Stamp Oct 31 2007
File Name \\ss7a.ds.man.ac.uk\vol5\vol3\users\snmrdata\2007\OCT07\OCT31\2201
Frequency (MHz) 299.85
Nucleus 1H
Number of Transients 16
Original Points Count 17962
Points Count 32768
Pulse Sequence s2pul
Receiver Gain 60.00
Solvent DEUTERIUM OXIDE
Spectrum Offset (Hz) 1799.0627
Sweep Width (Hz) 4797.60
Temperature (degree C) 29.000
2201
VerticalScaleFactor = 1
Normalized Intensity
Chemical Shift (ppm)
